# Supplementary material for: An octameric PqiC toroid stabilises the outer-membrane interaction of the PqiABC transport system
Source: EMBO Rep. 2024 Jan 16;25(1):82–101. doi: 10.1038/s44319-023-00014-4 (PMC10897342; doi:10.1038/s44319-023-00014-4)
Supplement: Supplementary file 1 — Appendix [file 44319_2023_14_MOESM1_ESM.docx]

**An Octameric PqiC toroid stabilises the outer-membrane interaction of the PqiABC transport system.**

Benjamin F Cooper^a^, Giedre Ratkeviciute^b^, Luke A Clifton^c^, Hannah Johnston^d^, Rachel Holyfield^d^, David J Hardy^d^, William Chatterton^d^, Pooja Sridhar^d^, Peter Wotherspoon^d^, Gareth W Hughes^e^, Simon G Caulton^d^, Stephen C L Hall^c,^ Andrew L Lovering^d^ and Timothy J Knowles^d*^.

**Appendix**

Table of Contents

[Appendix Figure S1 - PqiB and LetB form homohexameric assemblies. 3](#_Toc147386038)

[Appendix Figure S2 - Proteins encoded by the Pqi and Let operons. 5](#_Toc147386039)

[Appendix Figure S3 – Purification of PqiC Constructs. 6](#_Toc147386040)

[Appendix Figure S4. – Interactions Stabilising the PqiC Interface. 8](#_Toc147386041)

[Appendix Figure S5 – Electrostatics of the PqiB:PqiC Interface as Predicted by AlphaFold-Multimer. 9](#_Toc147386042)

[Appendix Figure S6 – Consurf Conservation Analysis of PqiC. 11](#_Toc147386043)

[Appendix Figure S7 – Confidence of the PqiB:PqiC Interface as predicated by AlphaFold-Multimer. 12](#_Toc147386044)

[Appendix Figure S8 – Comparison of the PqiC and PqiC^17-187^ X-ray Structures. 14](#_Toc147386045)

[Appendix Figure S9 – QCM-D Control Experiments. 15](#_Toc147386046)

[Appendix Figure S10 – Purification of PqiAB. 16](#_Toc147386047)

[Appendix Figure S11 – Phenotypic Complementation Replicates. 17](#_Toc147386048)

[Appendix Figure S12 – PqiABC knockout generation and Phenotypic Complementation Western Blot. 18](#_Toc147386049)

[Appendix Table S1 – Data Collection and Refinement Statistics for the PqiC Structures. 19](#_Toc147386050)

[Appendix Table S2 – PqiC Interface Interactions as Identified by PDBePISA. 21](#_Toc147386051)

[Appendix Table S3 – Neutron Reflectometry Fit Parameters. 22](#_Toc147386052)

[Appendix Table S4 – Oligonucleotides Utilised in This Study. 23](#_Toc147386053)

[Appendix Table S5 – Plasmids Utilised in This Study. 25](#_Toc147386054)

[Appendix Table S6 – Strains Utilised in This Study. 26](#_Toc147386055)

# Appendix Figure S1 - PqiB and LetB Form Homohexameric Assemblies.


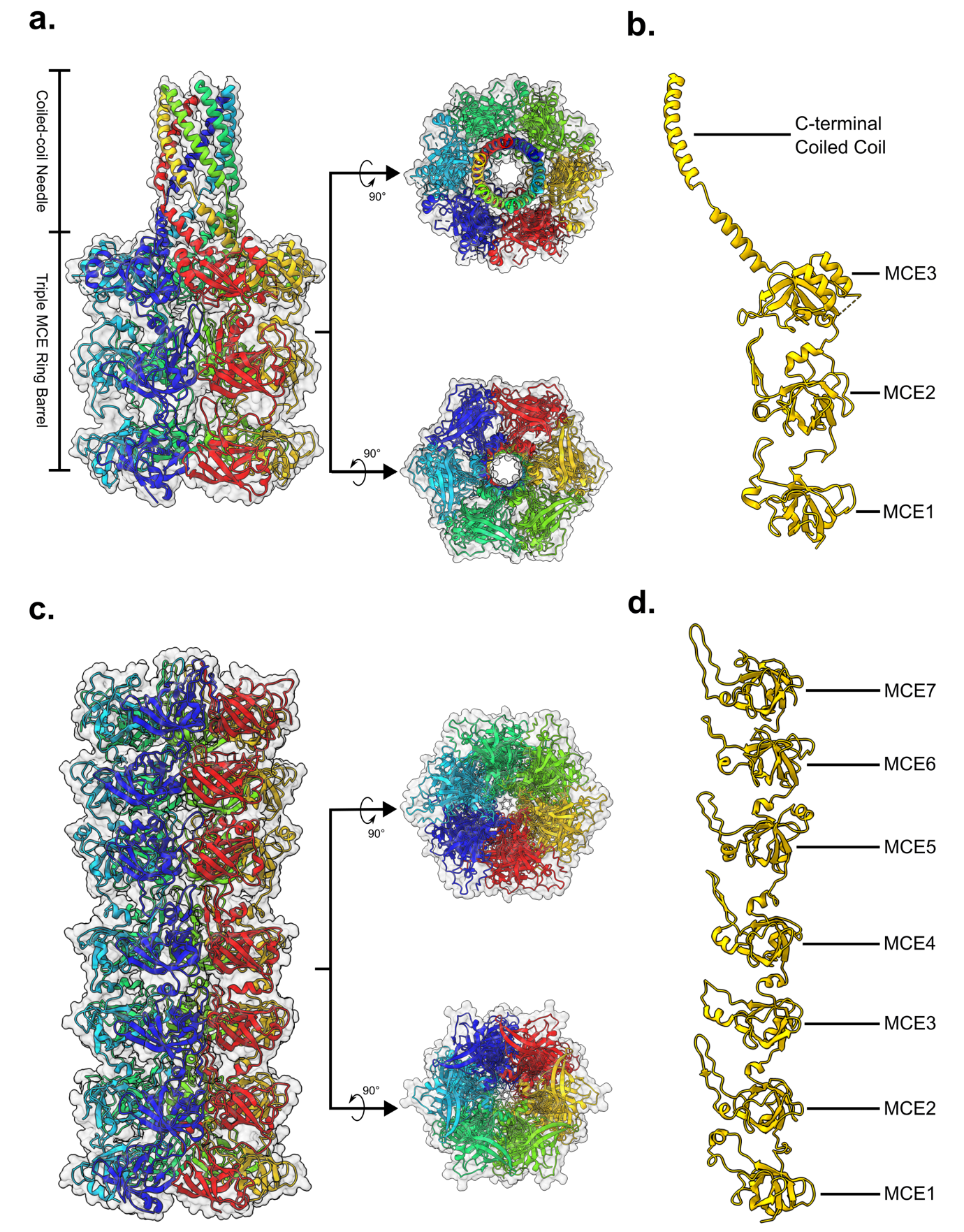


**Appendix Figure S1 - PqiB and LetB form Homohexameric Assemblies.**

a) Orthogonal views of PqiB residues 39-431 (PDB: 5UVN), depicted as a cartoon with rainbow chain colouring and a transparent grey molecular surface, highlighting the hexameric nature of its quaternary structure and the presence of a central channel. b) Cartoon representation of residues 39-431 of a single PqiB monomer. c) Orthogonal views of LetB residues 46-877 (PDB: 6V0C), depicted as a cartoon with rainbow chain colouring and a transparent grey molecular surface, highlighting the hexameric nature of its quaternary structure and the presence of a central channel. d) Cartoon representation of residues 46-877 of a single LetB monomer.

# Appendix Figure S2 - Proteins Encoded by the Pqi and Let Operons.


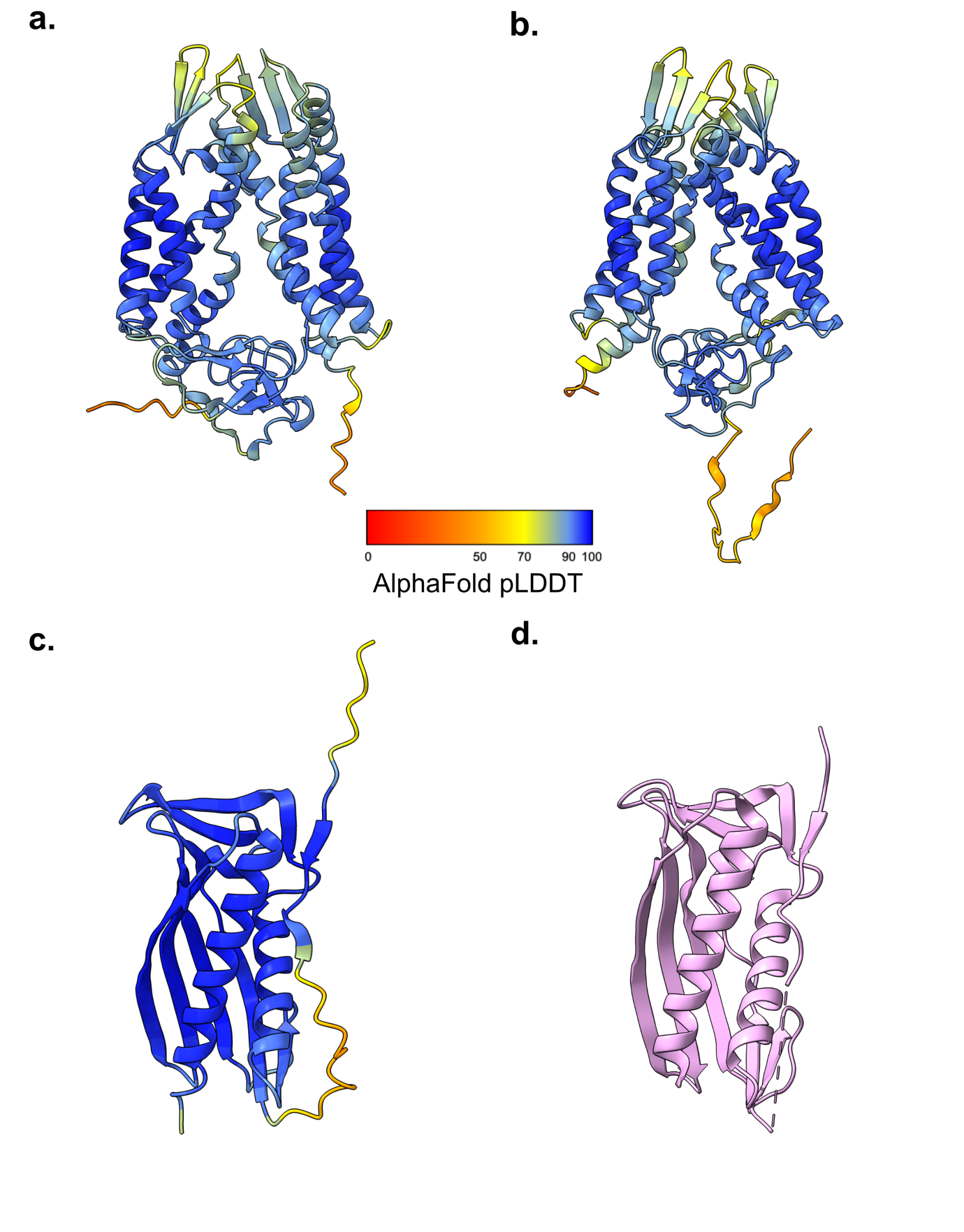


**Appendix S2 – Proteins Encoded by the Pqi and Let (formerly Yeb) Operons.**

a) AlphaFold predicted structure of PqiA (AF-P0AFL9-F1) coloured according to pLDDT. b) AlphaFold predicted structure of LetA/YebS (AF-P0AD03-F1) coloured according to pLDDT. c) b) AlphaFold predicted structure of PqiC residues 16-187 (AF-P0AB10-F1) coloured according to pLDDT. d) Crystal structure of *Enterobacter cloacae* PqiC (PDB: 6OSX). An AlphaFold pLDDT colour key is shown in the centre of the figure.

# Appendix Figure S3 – Purification of PqiC Constructs.


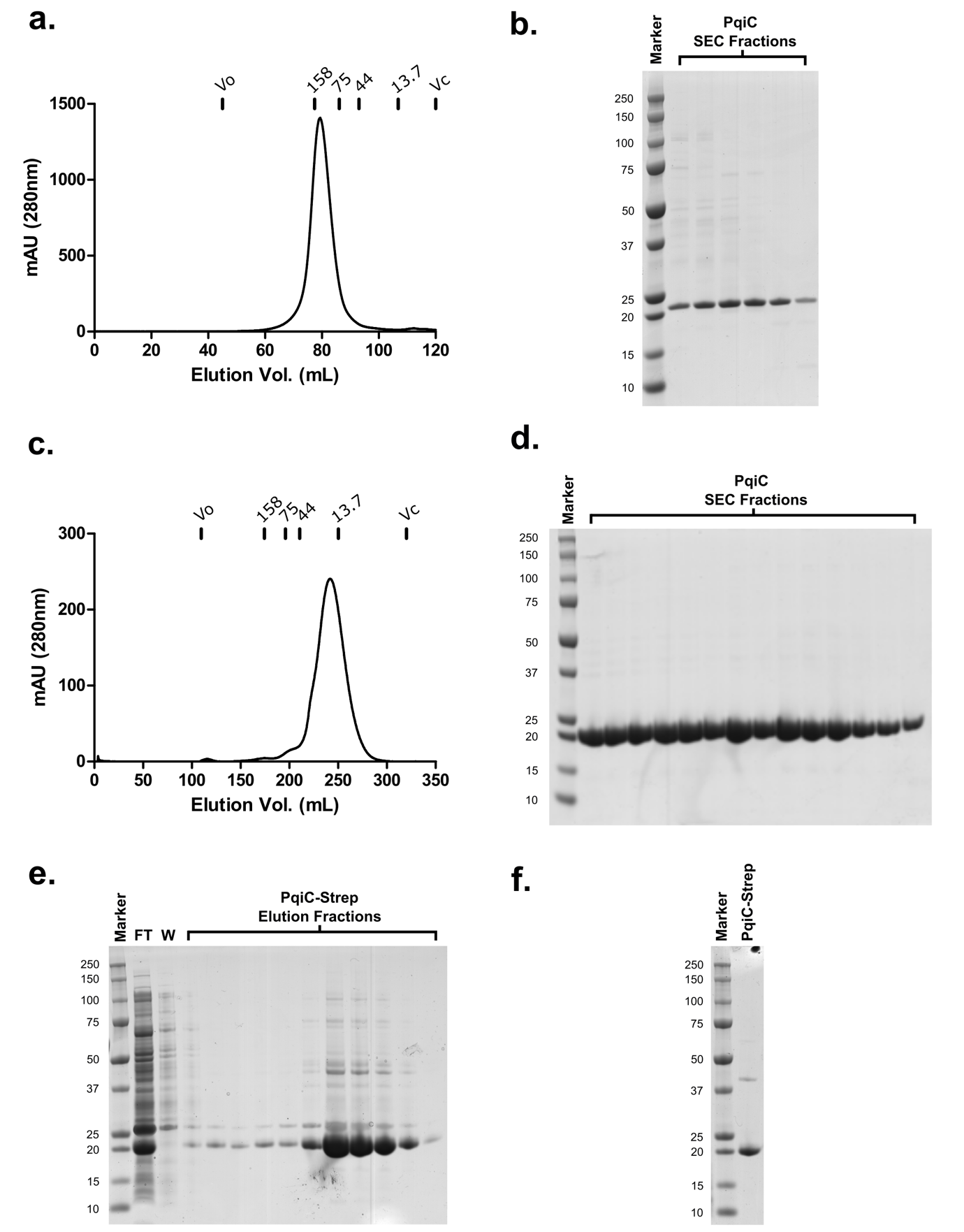


**Appendix Figure S3 - Purification of PqiC constructs.**

a) Superdex 200 16/60 SEC trace of PqiC showing a single elution peak after the void peak. Molecular weight marker positions are indicated at the top of the trace. b) SDS-PAGE of PqiC SEC fractions show a predominant band between 20 and 25 kDa with minor contaminants at higher molecular masses. c) Superdex 200 26/600 SEC trace of PqiC^17-187^ showing a single elution peak. Molecular weight marker positions are indicated at the top of the trace. d) SDS-PAGE of PqiC^17-187^ SEC fractions showing a single band at 20 kDa with no major contaminants. e) PqiC Strep TagII purification indicating the flowthrough - FT, wash - W and elution fractions. f) PqiC Strep Tag II following PD-10 mediated buffer exchange into 50 mM Tris, 150 mM NaCl, 0.05 % w/v n-dodecyl-β-D-maltoside; pH 8.

# Appendix Figure S4. – Interactions Stabilising the PqiC Interface.


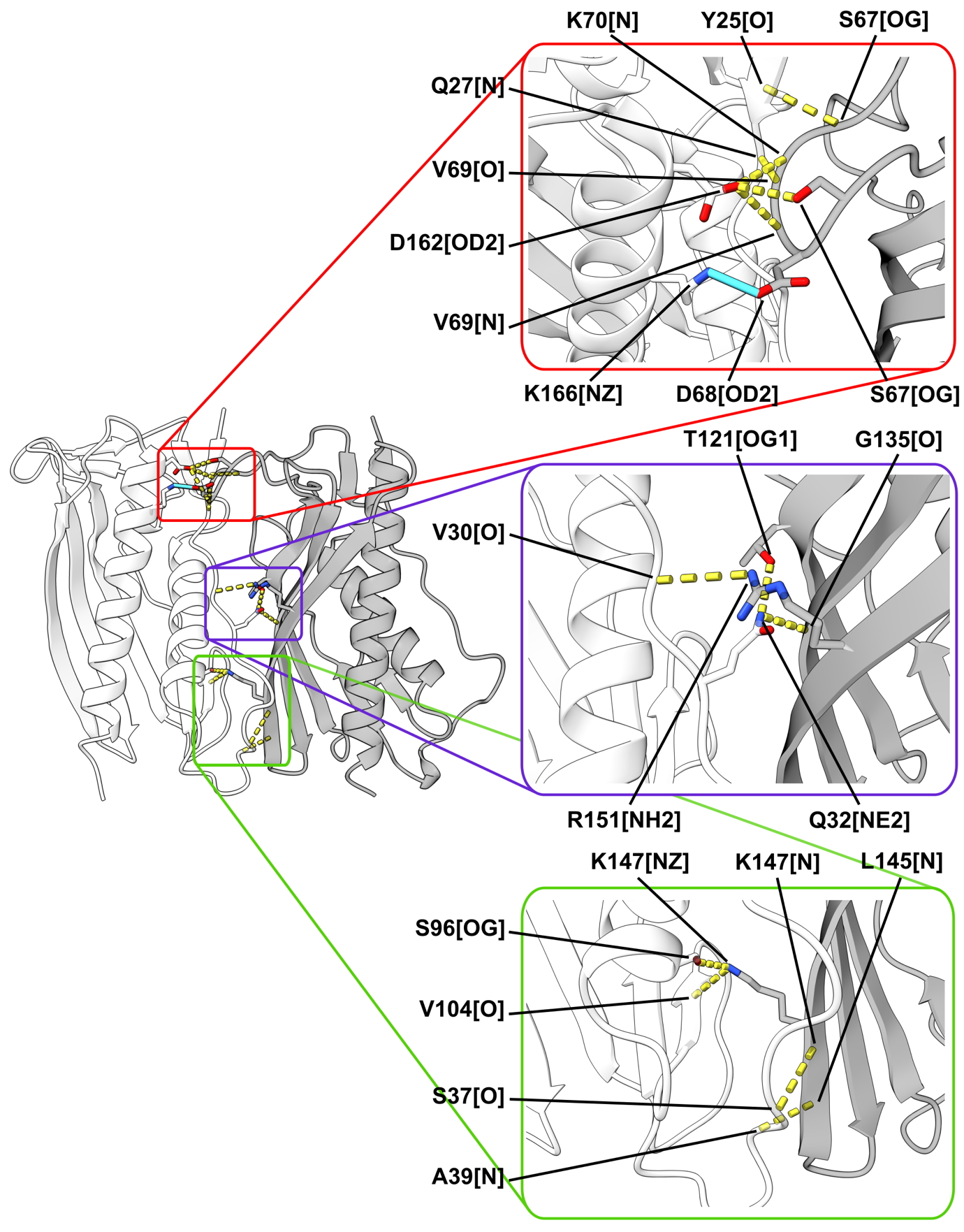


**Appendix Figure S4 – Interactions Stabilising the PqiC Interface.**

Cartoon representation of the PqiC structure with the two chains coloured in white and grey. Panels to the right indicate the interactions between the monomers as identified by PDBePSIA. Hydrogen bonds and salt bridges are indicated in yellow and cyan respectively. Atoms involved in these interactions are labelled accordingly.

# Appendix Figure S5 – Electrostatics of the PqiB:PqiC Interface as Predicted by AlphaFold-Multimer.


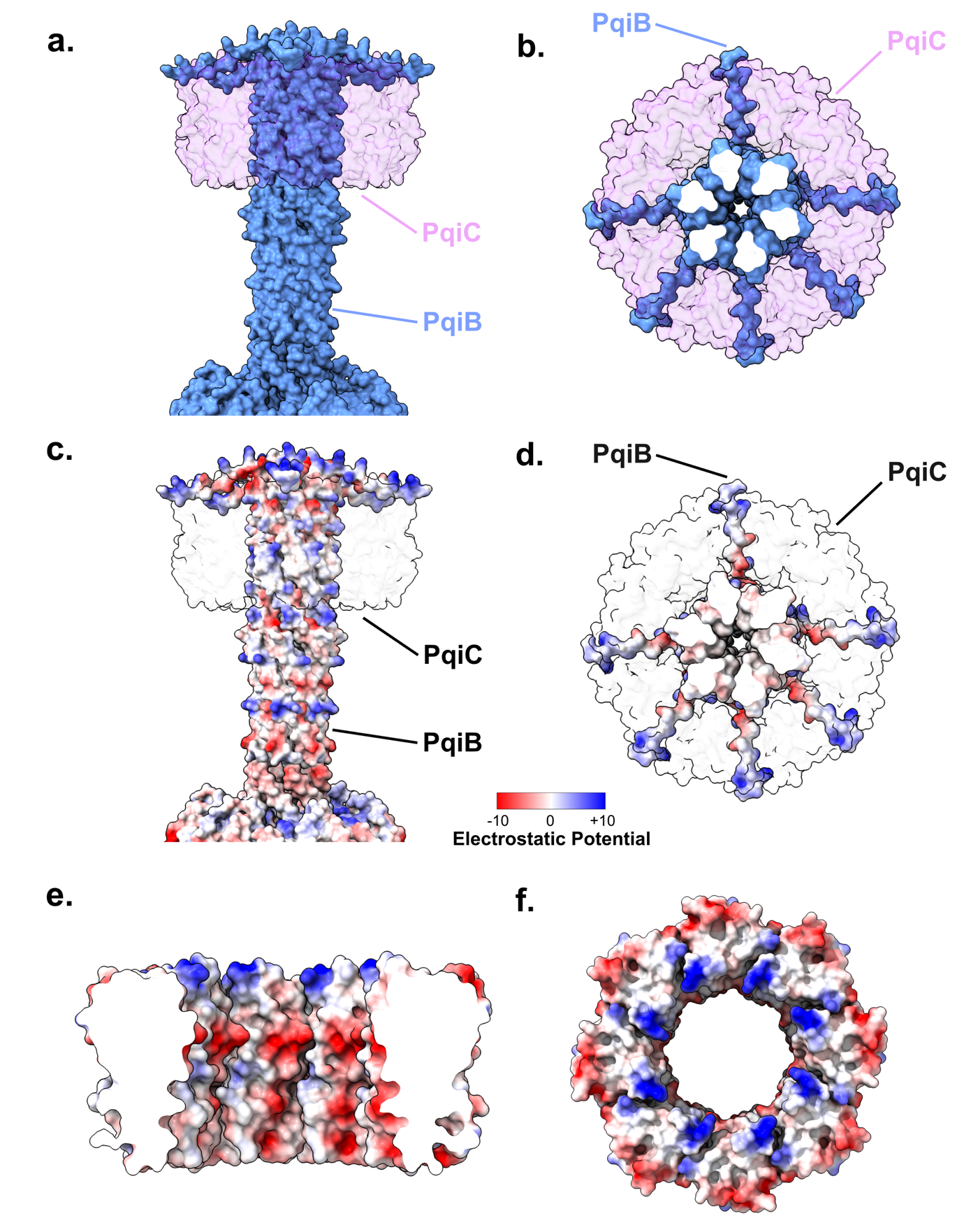


**Appendix Figure S5 – Electrostatics of the PqiB:PqiC Interface as predicted by AlphaFold-Multimer.**

a) Side view of the AlphaFold-Multimer prediction for the PqiB-PqiC interaction. Proteins are depicted as surface representations coloured in blue and purple for PqiB and PqiC respectively. b) Clipped view from the base of the PqiB coiled-coil of the AlphaFold-Multimer prediction for the PqiB-PqiC interaction. Proteins are depicted as surface representations coloured in blue and purple for PqiB and PqiC respectively. c) Side view of the AlphaFold-Multimer prediction for the PqiB-PqiC interaction. The surface electrostatic potential is displayed for PqiB, indicating no clear reciprocal regions of charge to those found in PqiC. PqiC is indicated as a transparent white surface. d) Clipped view from the base of the PqiB coiled-coil of the AlphaFold-Multimer prediction for the PqiB-PqiC interaction. The surface electrostatic potential is displayed for PqiB, indicating potential complementary charged regions, at its extreme C-terminus, to those upon the membrane occluded face of PqiC. e) Surface representation of the PqiC pore lining coloured according to electrostatic potential indicating the distinct regions of negative charge. f) Surface representation of the PqiC octamer, viewed from the membrane plane, coloured according to electrostatic potential demonstrating its radial polarisation.

# Appendix Figure S6 – Consurf Conservation Analysis of PqiC.


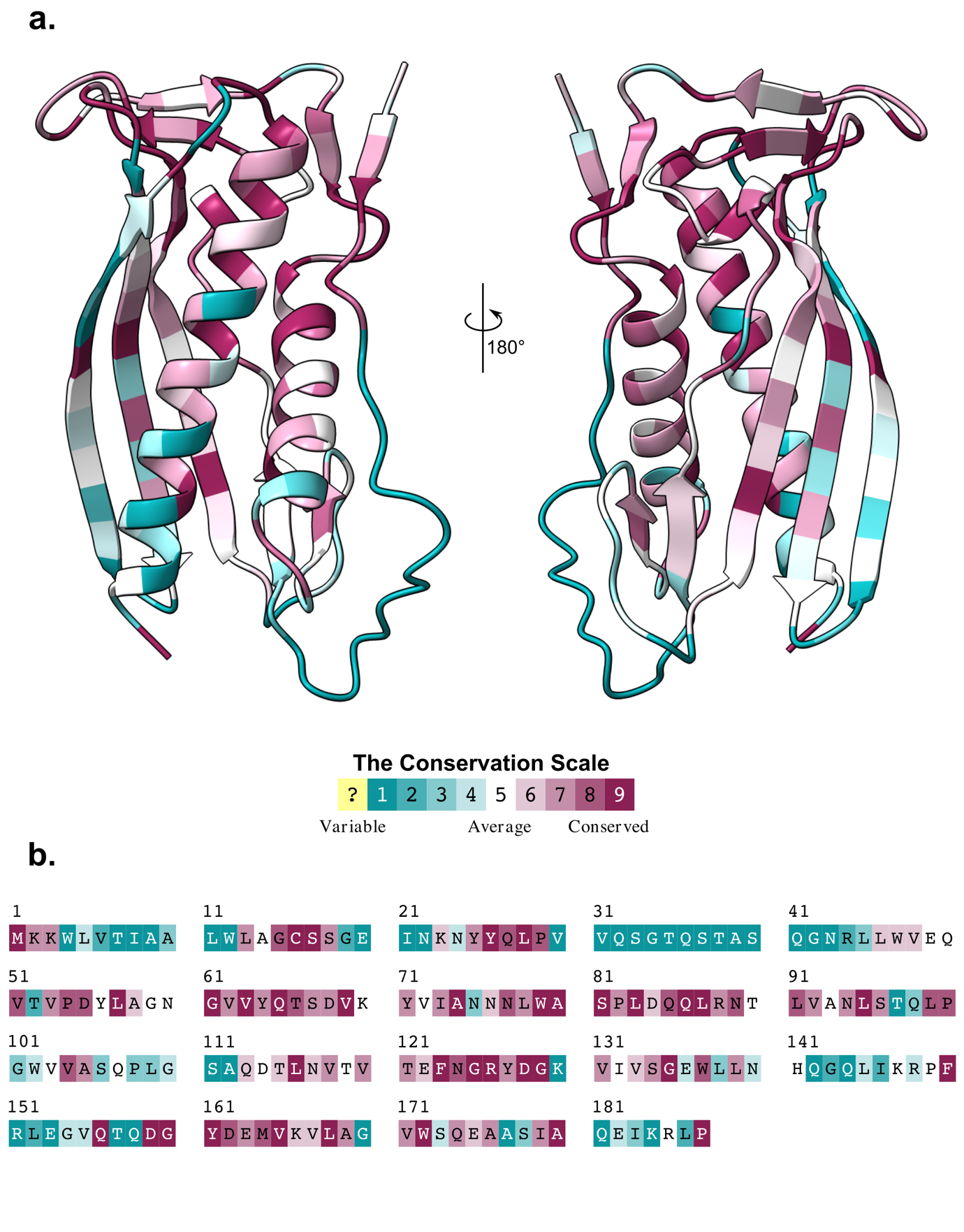
**Appendix Figure S6 - Consurf conservation analysis of PqiC.**

a) Orthogonal views of PqiC structure chain B displayed as cartoon representation with residues coloured according to Consurf sequence conservation. (B) PqiC sequence coloured according to Consurf sequence conservation. The Consurf conservation scale is included at the bottom centre of the figure.

# Appendix Figure S7 – Confidence of the PqiB:PqiC Interface as predicated by AlphaFold-Multimer.


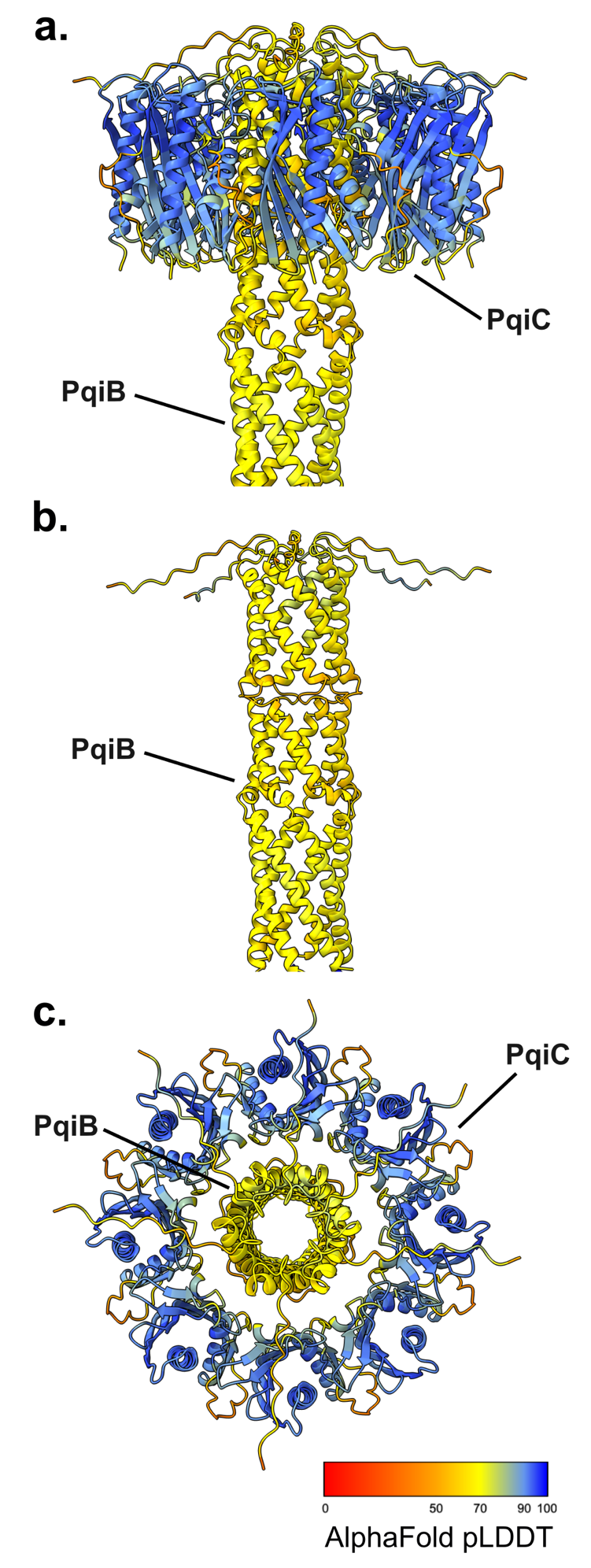


**Appendix Figure S7 – Confidence of the PqiB:PqiC Interface as predicted by AlphaFold-Multimer.**

a) Side view of the PqiB-PqiC interface as predicated by AlphaFold-Multimer with proteins coloured according to the pLDDT score. b) Side view of the PqiB moiety of the AlphaFold-Multimer PqiB-PqiC interaction coloured according to pLDDT score. c) Top view of the PqiB-PqiC interface as predicated by AlphaFold-Multimer with proteins coloured according to the pLDDT score. An AlphaFold pLDDT colour key is shown at the base of the figure.

# Appendix Figure S8 – Comparison of the PqiC and PqiC^17-187^ X-ray Structures.


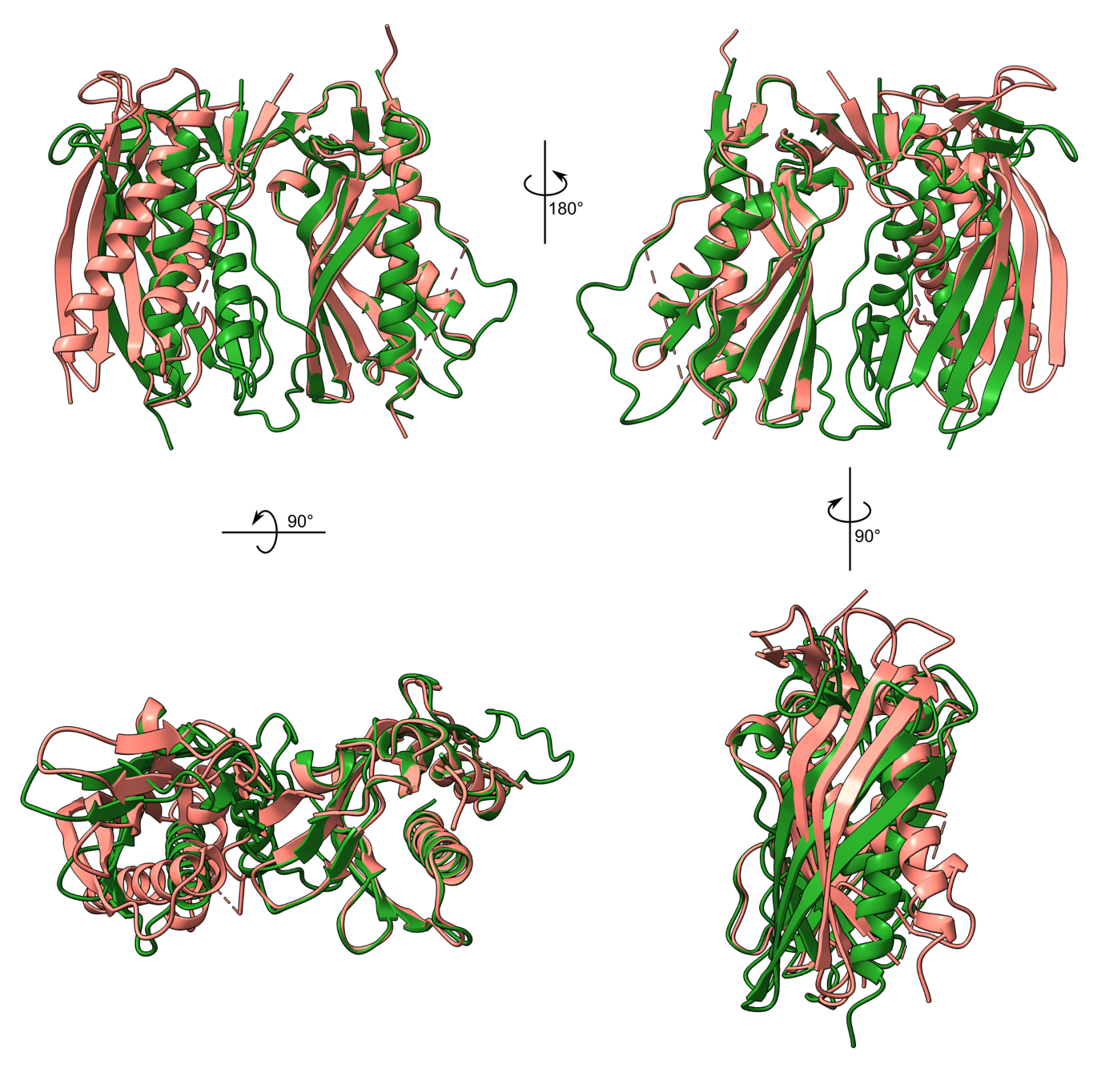


**Appendix Figure S8 - Comparison of the PqiC and PqiC^17-187^ X-ray structures.**

Overlay of the aligned PqiC (green) and PqiC^17-187^ (salmon) indicating the differing geometries of the A and B chains in both structures. Note Chain C from the PqiC^17-187^ structure has been removed for clarity.

# Appendix Figure S9 – QCM-D Control Experiments.


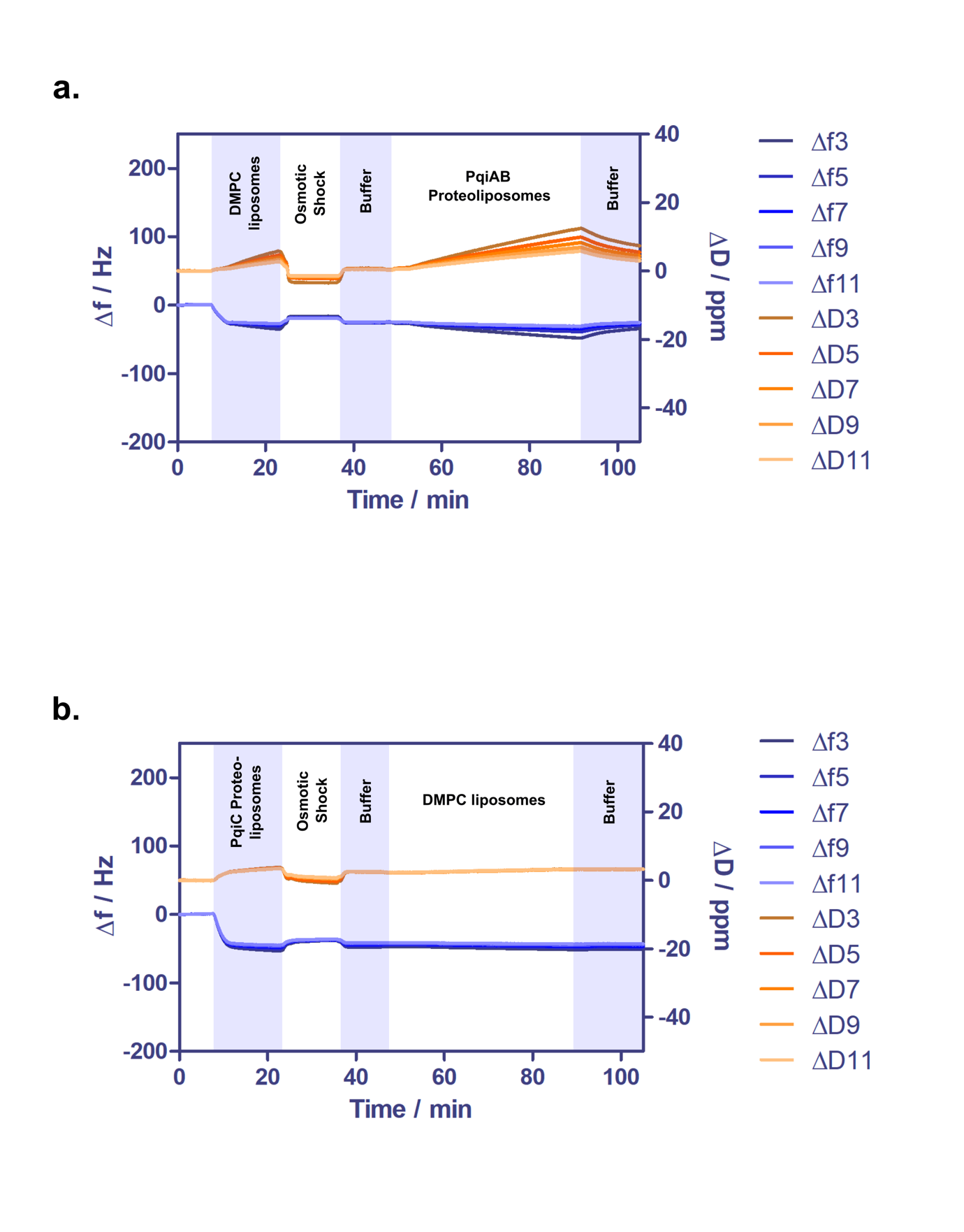


**Appendix Figure S9 - QCM-D control experiments.**

a). Deposition of a DMPC bilayer followed by subsequent addition of PqiAB-proteoliposomes showing a weak interaction with the membrane (~-20Hz). Subsequent washing with buffer was sufficient to dislodge and remove bound PqiAB-proteoliposomes. b) Deposition of PqiC-proteoliposomes to form a PqiC tethered planar bilayer with subsequent addition of DMPC liposomes showing no interaction taking place.

# Appendix Figure S10 – Purification of PqiAB.


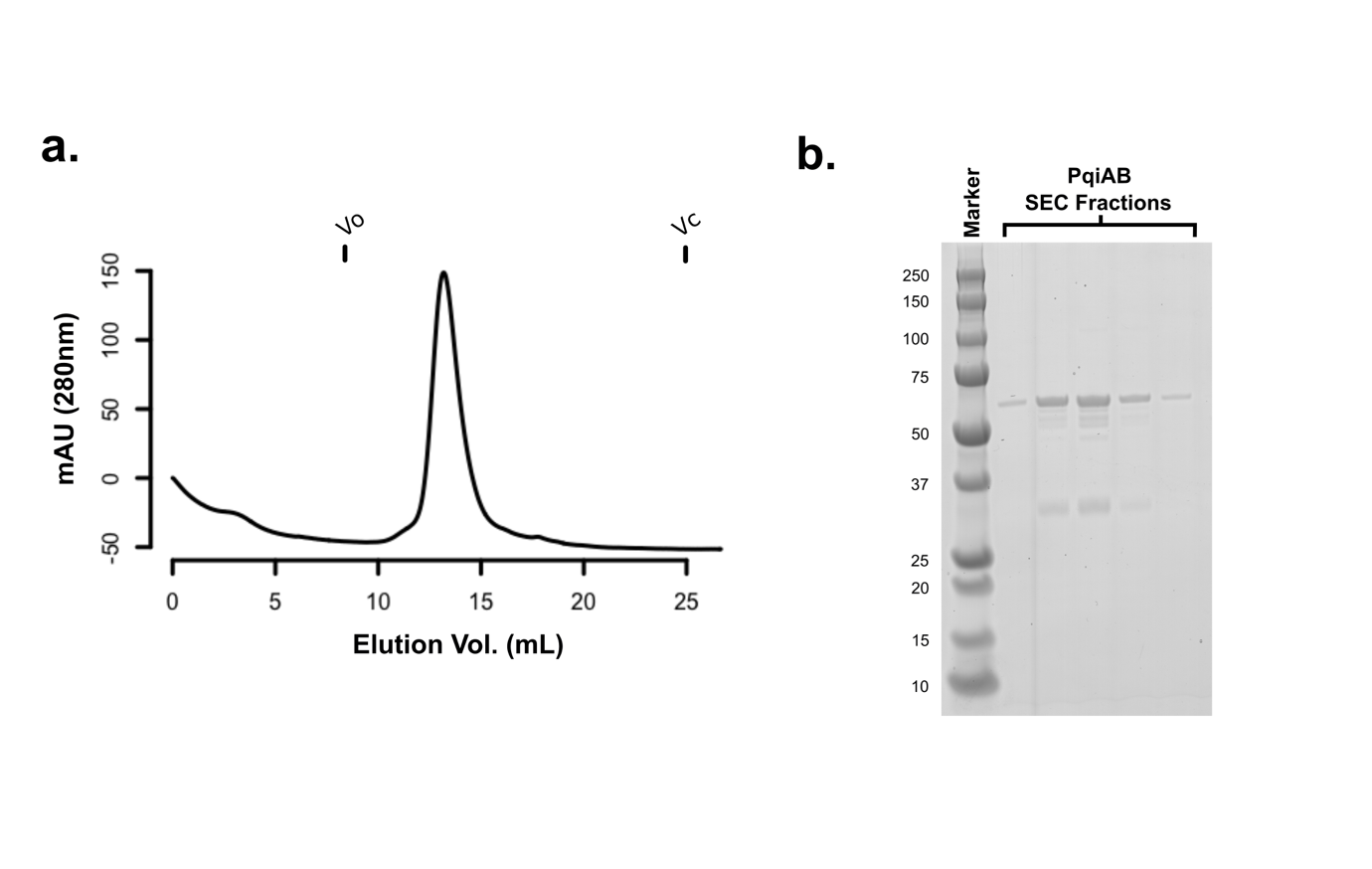


**Appendix Figure S10 - Purification of PqiAB.**

a) Superose 6 10/300 Increase SEC trace of PqiAB showing a single elution peak at approximately 13 ml. b) SDS-PAGE of PqiAB SEC fractions showing two predominant bands at 35 kDa and 60 kDa corresponding to PqiA and PqiB respectively.

Appendix Figure S11 – Phenotypic Complementation Replicates.


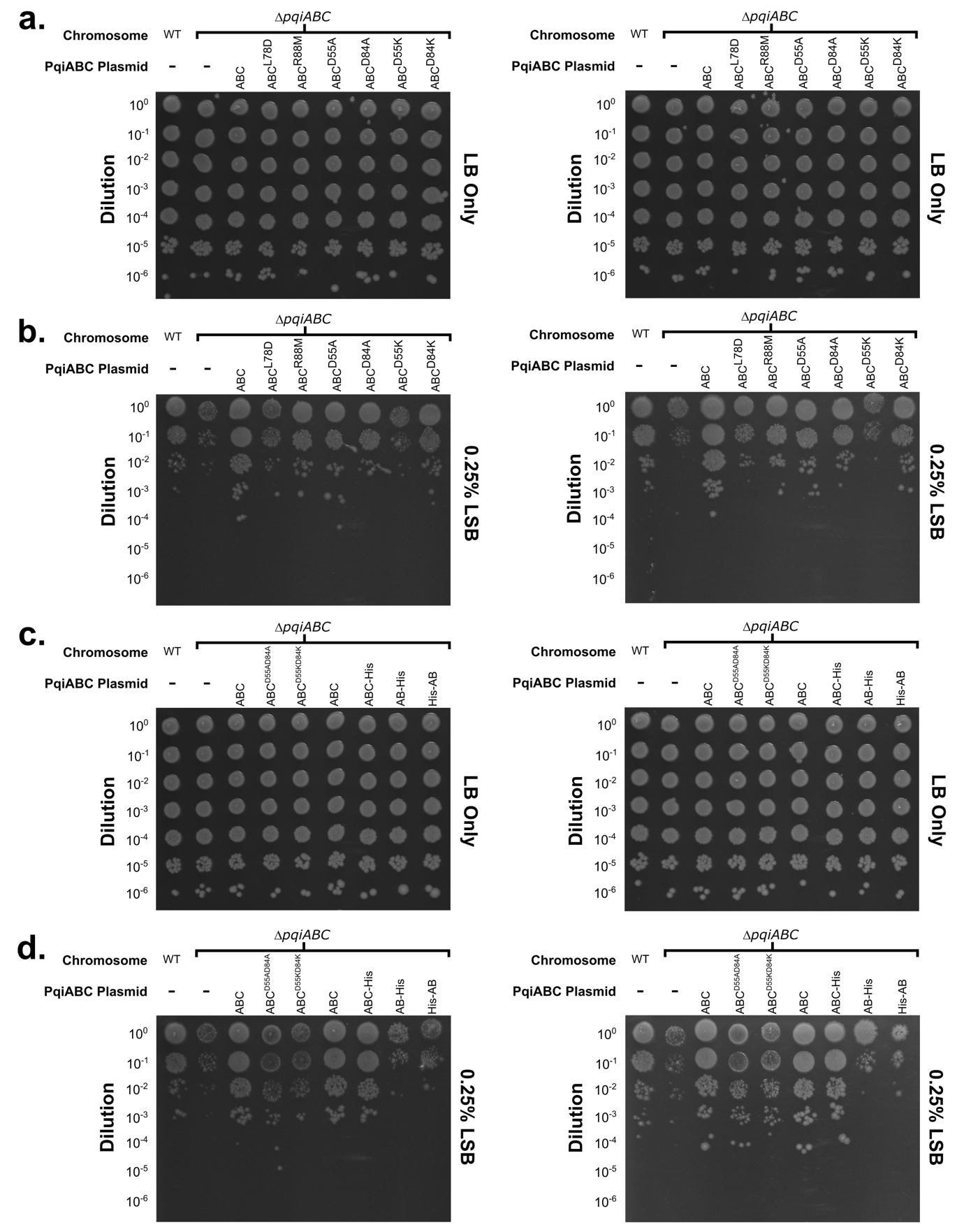


**Appendix Figure S11 - Phenotypic complementation replicates.**

a) Replicates for the growth of the single PqiC mutant strains on LB only. b) Replicates for the growth of the single PqiC mutant strains on LB supplemented with 0.25% LSB. c) Replicates for the growth of the double PqiC mutant and PqiAB strains on LB only. d) Replicates for the growth of the double PqiC mutant and PqiAB strains on LB supplemented with 0.25% LSB.

# Appendix Figure S12 – PqiABC knockout generation and Phenotypic Complementation Western Blot.


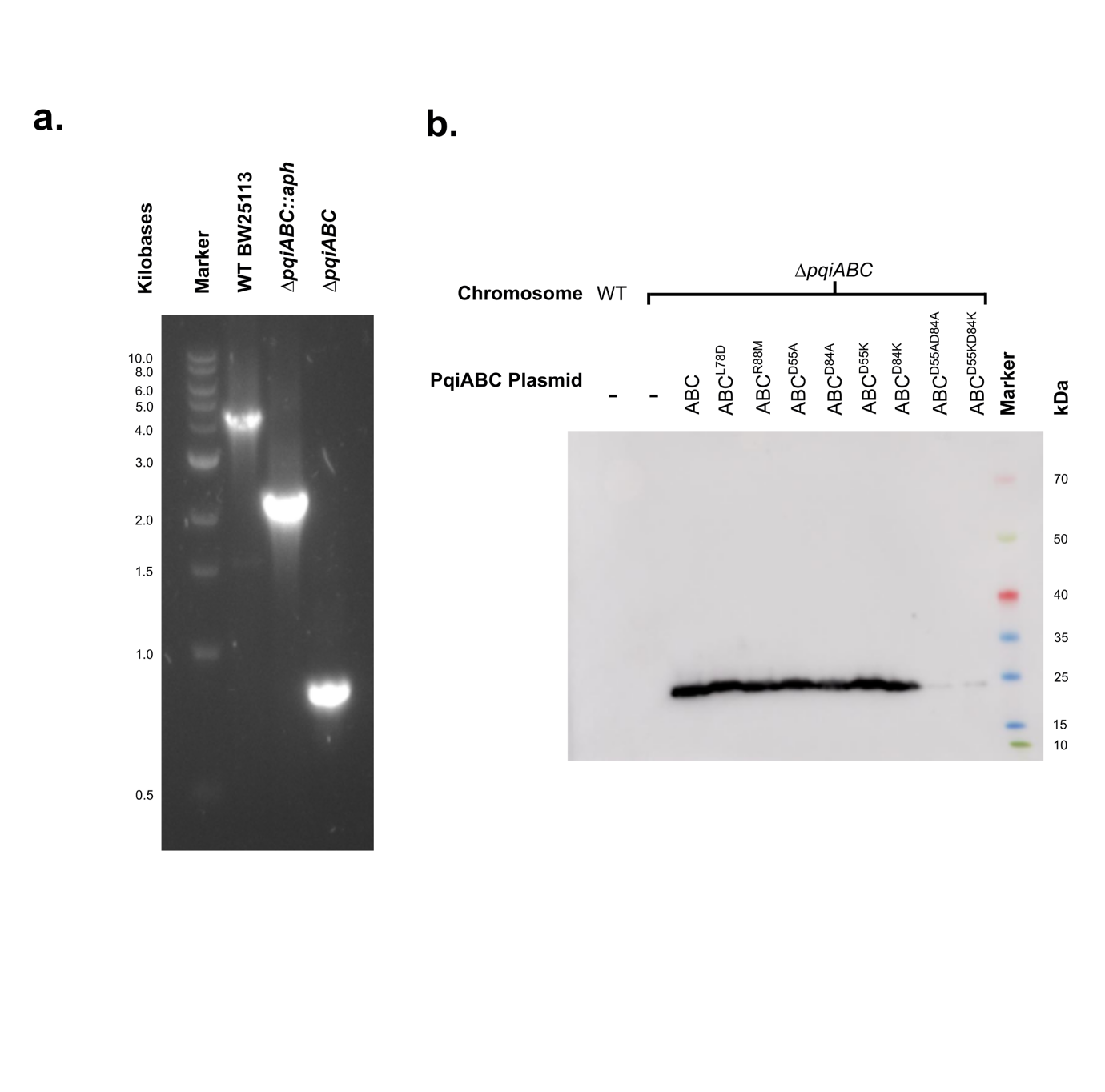


**Appendix Figure S12 - PqiABC knockout generation and phenotypic complementation western blot.**

a) Gel electrophoresis of colony PCR products confirming the generation of the *∆pqiABC*  strain. Lan 1- marker, Lane 2 – WT BW 25113 (amplification of the WT *pqi* operon), Lane 3 - BW25113 *∆ABC::aph* (amplification of the *aph* cassette in place of the *pqi* operon), lane 4 - BW25113 *∆pqiABC* (amplification of the scar region following *aph* cassette removal). b) Anti-His western blot of the strains utilised during the phenotypic complementation assays. No bands are present for the WT BW25113 (lane 1) or BW25113 ∆*pqiABC* (lane 2) strains due to absence of a PqiABC expressing plasmid. Bands at ~20 kDa indicate the presence of PqiC - the last gene in the *pqi* operon and site of all our investigated mutations. Bands were of a comparable intensity for all but the two double mutants (D55AD84A and D55KD84K, lanes 10 and 11) were distinctly lower, suggesting these PqiC mutants to be less stable within the cell.

# Appendix Table S1 – Data Collection and Refinement Statistics for the PqiC Structures.

|  | **PqiC** | **PqiC^17-187^** |
| --- | --- | --- |
| Wavelength | 0.9797 Å | 1.0000 Å |
| Resolution range | 53.77 - 3.211 (3.326 - 3.211) | 38.57 - 2.081 (2.155 - 2.081) |
| Space group | I 4 2 2 | P 61 |
| Unit cell | 132.148 132.148 185.043  90 90 90 | 133.603 133.603 65.715 90 90 120 |
| Total reflections | 8788 (132) | 67192 (450) |
| Unique reflections | 4394 (66) | 33852 (225) |
| Multiplicity | 2.0 (2.0) | 2.0 (2.0) |
| Spherical Completeness (%) | 31.81 (4.96) | 84.00 (5.60) |
| Elliptical Completeness (%) | 90.6 (72.4) | 95.7 (56) |
| Mean I/sigma(I) | 6.86 (1.97) | 18.79 (1.24) |
| Wilson B-factor | 72.82 | 46.75 |
| R-merge | 0.1247 (0.3041) | 0.02414 (0.5142) |
| R-meas | 0.1763 (0.4301) | 0.03414 (0.7272) |
| R-pim | 0.1247 (0.3041) | 0.02414 (0.5142) |
| CC1/2 | 0.977 (0.787) | 0.999 (0.613) |
| CC* | 0.994 (0.938) | 1 (0.872) |
| Reflections used in refinement | 4373 (66) | 33847 (225) |
| Reflections used for R-free | 217 (2) | 1683 (11) |
| R-work | 0.2469 (0.2458) | 0.2041 (0.3473) |
| R-free | 0.3049 (0.2251) | 0.2312 (0.4761) |
| CC(work) | 0.863 (0.873) | 0.955 (0.785) |
| CC(free) | 0.712 (1.000) | 0.933 (0.818) |
| Number of non-hydrogen atoms | 2582 | 3955 |
| Macromolecules | 2582 | 3640 |
| Ligands | 0 | 0 |
| Solvent | 0 | 315 |
| Protein residues | 330 | 461 |
| RMS(bonds) | 0.003 | 0.005 |
| RMS(angles) | 0.50 | 0.80 |
| Ramachandran favoured (%) | 95.40 | 99.33 |
| Ramachandran allowed (%) | 4.60 | 0.67 |
| Ramachandran outliers (%) | 0.00 | 0.00 |
| Rotamer outliers (%) | 0.00 | 0.75 |
| Clashscore | 8.77 | 4.69 |
| Average B-factor | 73.54 | 52.89 |

Statistics in parenthesis refer to the highest resolution shell.

#

# Appendix Table S2 – PqiC Interface Interactions as Identified by PDBePISA.

| **Interaction** | **Atom 1** | **Atom 2** | **Interaction Distance**  **(Å)** |
| --- | --- | --- | --- |
| Salt Bridge | B:LYS 166 [NZ] | A:ASP 68 [OD2] | 2.57 |
| Hydrogen Bond | B:GLN  27[ N  ] | A:VAL  69[ O  ] | 3.37 |
| Hydrogen Bond | B:GLN  32[ NE2] | A:THR 121[ OG1] | 2.93 |
| Hydrogen Bond | B:GLN  32[ NE2] | A:GLY 135[ O  ] | 3.15 |
| Hydrogen Bond | B:ALA  39[ N  ] | A:LEU 145[ O  ] | 2.46 |
| Hydrogen Bond | B:ASP 162[ OD2] | A:SER  67[ OG ] | 3.30 |
| Hydrogen Bond | B:ASP 162[ OD2] | A:VAL  69[ N  ] | 3.73 |
| Hydrogen Bond | B:ASP 162[ OD2] | A:LYS  70[ N  ] | 3.90 |
| Hydrogen Bond | B:TYR  25[ O  ] | A:TYR  71[ N  ] | 3.30 |
| Hydrogen Bond | B:SER  37[ O  ] | A:LYS 147[ N  ] | 3.65 |
| Hydrogen Bond | B:SER  96[ OG ] | A:LYS 147[ NZ ] | 2.26 |
| Hydrogen Bond | B:VAL 104[ O  ] | A:LYS 147[ NZ ] | 2.33 |
| Hydrogen Bond | B:VAL  30[ O  ] | A:ARG 151[ NH2] | 3.49 |

# Appendix Table S3 – Neutron Reflectometry Fit Parameters.

| **Parameter** | **Mean** | **Relative Low Error** | **Relative High Error** |
| --- | --- | --- | --- |
| Substrate Roughness (Å) | 8.5126 | 0.69347 | 0.65575 |
| SiO_2_ Thickness (Å) | 9.8881 | 1.2848 | 1.4255 |
| SiO_2_ SLD (Å^-2^) | 5.2028 | 0.60613 | 0.66772 |
| SiO_2_ Roughness (Å) | 4.5073 | 1.1829 | 1.1471 |
| Lipid APM (Å^2^) | 62.596 | 1.6648 | 1.9391 |
| Head Bound Waters | 5.7557 | 1.493 | 1.2716 |
| Bilayer Roughness (Å) | 5.2709 | 0.33195 | 0.34509 |
| SiO_2_ Hydration (%) | 36.344 | 5.4812 | 5.5895 |
| Protein Layer Thickness (Å) | 52.526 | 4.1976 | 4.1618 |
| Protein Layer Hydration (%) | 88.2 | 1.2383 | 1.1949 |
| Protein Layer Roughness (Å) | 6.6499 | 1.1347 | 1.0527 |
| Protein HG VF (%) | 18.43 | 8.3716 | 12.065 |
| Bilayer Coverage (%) | 95.363 | 1.5872 | 1.4694 |
| Protein Coverage (%) | 11.799 | 1.1949 | 1.2381 |

# Appendix Table S4 – Oligonucleotides Utilised in This Study.

| **Name** | **Sequence** | **Description** |
| --- | --- | --- |
| pqiA_ko_Fwd | AGTCATCTGTAAAATAGCGCATCATTAAGGAGTACCAATGGTGTAGGCTGGAGCTGCTTC | Forward primer for the amplification of the pKD4 kanamycin cassette for the *pqiABC* knockout. |
| pqiC_ko_Rev | TATTTACAAATCTTTATAATTAAGGTAGACGCTTTATCTCCATATGAATATCCTCCTTAG | Reverse primer for the amplification of the pKD4 kanamycin cassette for *the* *pqiABC* knockout. |
| delABC_comf_Fwd | GAAGCCCTACAGACGCAAGT | Forward primer for colony PCR to confirm disruption off the *pqiABC* genes. |
| delABC_comf_Rev | CCATTACTACCCTGTCCGCC | Reverse primer for colony PCR to confirm disruption off the *pqiABC* genes. |
| PqiC L78D Fwd | CAACAACAACgatTGGGCCAGCCC | Forward primer to introduce the PqiC L78D mutation into the PqiABC construct |
| PqiC L78D Rev | GCAATCACATACTTCACATC | Reverse primer to introduce the PqiC L78D mutation into the PqiABC construct |
| PqiC D55A Fwd | CACTGTTCCTgcgTATCTGGCGG | Forward primer to introduce the PqiC D55A mutation into the PqiABC construct |
| PqiC D55A Rev | ACCTGCTCTACCCATAAC | Reverse primer to introduce the PqiC D55A mutation into the PqiABC construct |
| PqiC D55K Fwd | CACTGTTCCTaaaTATCTGGCGG | Forward primer to introduce the PqiC D55K mutation into the PqiABC construct |
| PqiC D55K Rev | ACCTGCTCTACCCATAAC | Reverse primer to introduce the PqiC D55K mutation into the PqiABC construct |
| PqiC D84A Fwd | CAGCCCGTTGgcgCAACAGTTGC | Forward primer to introduce the PqiC D84A mutation into the PqiABC construct |
| PqiC D84A Rev | GCCCACAAGTTGTTGTTG | Reverse primer to introduce the PqiC D84A mutation into the PqiABC construct |
| PqiC D84K Fwd | CAGCCCGTTGaaaCAACAGTTGC | Forward primer to introduce the PqiC D84K mutation into the PqiABC construct |
| PqiC D84K Rev | GCCCACAAGTTGTTGTTG | Reverse primer to introduce the PqiC D84K mutation into the PqiABC construct |
| PqiC R88M Fwd | TCAACAGTTGatgAACACCCTGGTTGCCAACCTGAGTACGC | Forward primer to introduce the PqiC R88M mutation into the PqiABC construct |
| PqiC R88M Rev | TCCAACGGGCTGGCCCAC | Reverse primer to introduce the PqiC R88M mutation into the PqiABC construct |
| PqiABC N-His Fwd | catcaccatTGCGAACATCATCATGCC | Forward primer to make the PqiABC N-ter tag |
| PqiABC N-His Rev | gtgatggtgCATATGTATATCTCCTTCTTAAAGTTAAAC | Reverse primer to make the PqiABC N-ter tag |
| PqiABC △C-His Fwd | TGAGATCCGGCTGCTAAC | Forward primer to introduce a stop codon after PqiC in the PqiABC construct |
| PqiABC △C-His Rev | AGGTAGACGCTTTATCTCTTG | Reverse primer to introduce a stop codon after PqiC in the PqiABC construct |
| PqiAB N-His1 Fwd | [CCATTGCGAACATCATCATGCC](https://www.thermofisher.com/order/catalog/product/10336022?SKULINK) | Forward primer to make the PqiAB N-ter tag - step 1 |
| PqiAB N-His1 Rev | [TGATGCATATGTATATCTCCTTCTTAAAGTTAAAC](https://www.thermofisher.com/order/catalog/product/10336022?SKULINK) | Reverse primer to make the PqiAB N-ter tag - step 1 |
| PqiAB N-His2 Fwd | [TCACTGCGAACATCATCATGCC](https://www.thermofisher.com/order/catalog/product/10336022?SKULINK) | Forward primer to make the PqiAB N-ter tag - step 2 |
| PqiAB N-His2 Rev | [TGGTGATGGTGATGCATATGTATATCTC](https://www.thermofisher.com/order/catalog/product/10336022?SKULINK) | Reverse primer to make the PqiAB N-ter tag - step 2 |
| PqiAB △C-His Fwd | [ACAACTCGAGTGACACCACCACCACCAC](https://www.thermofisher.com/order/catalog/product/10336022?SKULINK) | Forward primer to introduce a stop codon after PqiB in the PqiAB construct |
| PqiAB △C-His Rev | [TTCGCCCTCTTCGGCTCT](https://www.thermofisher.com/order/catalog/product/10336022?SKULINK) | Reverse primer to introduce a stop codon after PqiB in the PqiAB construct |

#

# Appendix Table S5 – Plasmids Utilised in This Study.

| **Construct Name** | **Description** | **Source** |
| --- | --- | --- |
| PqiC | Expression of PqiC 1-187 with C-terminal hexa-histidine tag | GenScript |
| PqiC-Strep | Expression of PqiC 1-187 with C-terminal StrepTag II tag | GenScript |
| PqiABC_Ntag | Expression of PqiABC with N-terminal hexa-histidine tag on PqiA | This Study |
| PqiAB_Ntag | Expression of PqiAB with N-terminal hexa-histidine tag on PqiA | This Study |
| PqiABC_Ctag | Expression of PqiABC with C-terminal hexa-histidine tag on PqiC | GenScript |
| PqiAB_Ctag | Expression of PqiAB with C-terminal hexa-histidine tag on PqiB | Genscript |
| pKD4 | Kanamycin resistance cassette flanked by FLP recombinase target sites. | (Datsenko and Wanner, 2000) |
| pCP20 | Temperature sensitive plasmid expressing FLP recombinase. | (Datsenko and Wanner, 2000) |
| PqiABC^L78D^ | PqiABC plasmid with L78D mutation in PqiC | This Study |
| PqiABC^D55A^ | PqiABC plasmid with D55A mutation in PqiC | This Study |
| PqiABC^D55K^ | PqiABC plasmid with D55K mutation in PqiC | This Study |
| PqiABC^D84A^ | PqiABC plasmid with D84A mutation in PqiC | This Study |
| PqiABC^D84K^ | PqiABC plasmid with D84K mutation in PqiC | This Study |
| PqiABC^R88M^ | PqiABC plasmid with R88M mutation in PqiC | This Study |
| PqiABC^D55AD84A^ | PqiABC plasmid with D55A D84A mutations in PqiC | This Study |
| PqiABC^D55KD84K^ | PqiABC plasmid with D55K D84K mutations in PqiC | This Study |

# Appendix Table S6 – Strains Utilised in This Study.

| **Strain** | **Usage** | **Source** |
| --- | --- | --- |
| E. coli K-12 BW25113 | Knockout generation and complementation studies | (Baba et al., 2006) |
| E. coli K-12 BW25113 ∆pqiABC::aph | Complementation Studies | This Study |
| E. coli K-12 BW25113 ∆pqiABC | Complementation Studies | This Study |
| E. coli C43 DE3 | Recombinant protein expression | (Miroux and Walker, 1996) |
| E. coli DH5ɑ | Plasmid replication (SDM) | New England Biolabs |
