# Supplementary figures and images for: An octameric PqiC toroid stabilises the outer-membrane interaction of the PqiABC transport system
Source: EMBO Rep. 2024 Jan 16;25(1):82–101. doi: 10.1038/s44319-023-00014-4 (PMC10897342; doi:10.1038/s44319-023-00014-4)

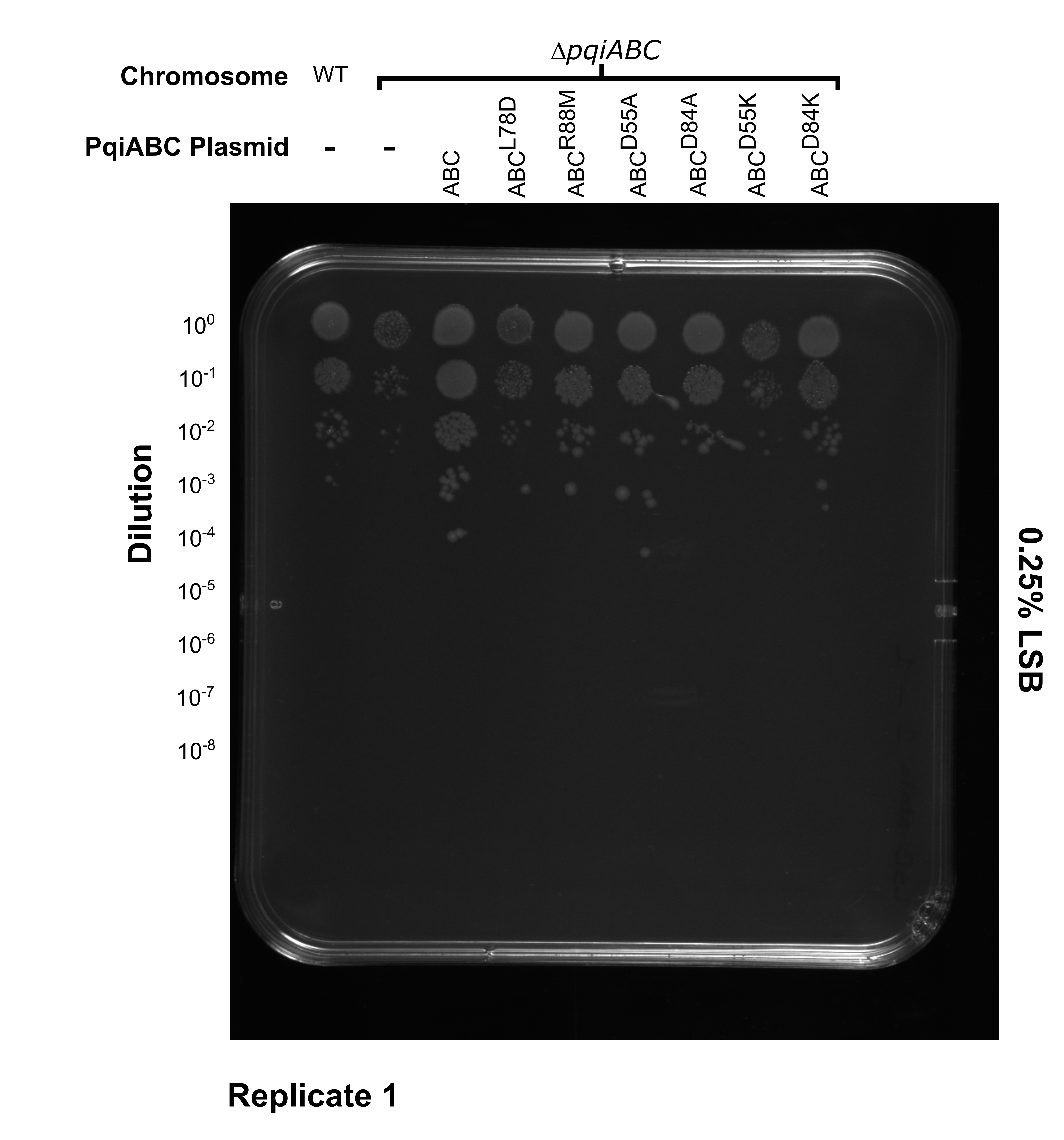

Supplement: Supplementary file 3 — Source Data Fig. 5 [file 44319_2023_14_MOESM3_ESM.zip › Source Data/Figure 5/5C/Figure 5C - 0.25% LSB replicates/Figure 5C - 0.25% LSB - Replicate 1.png]

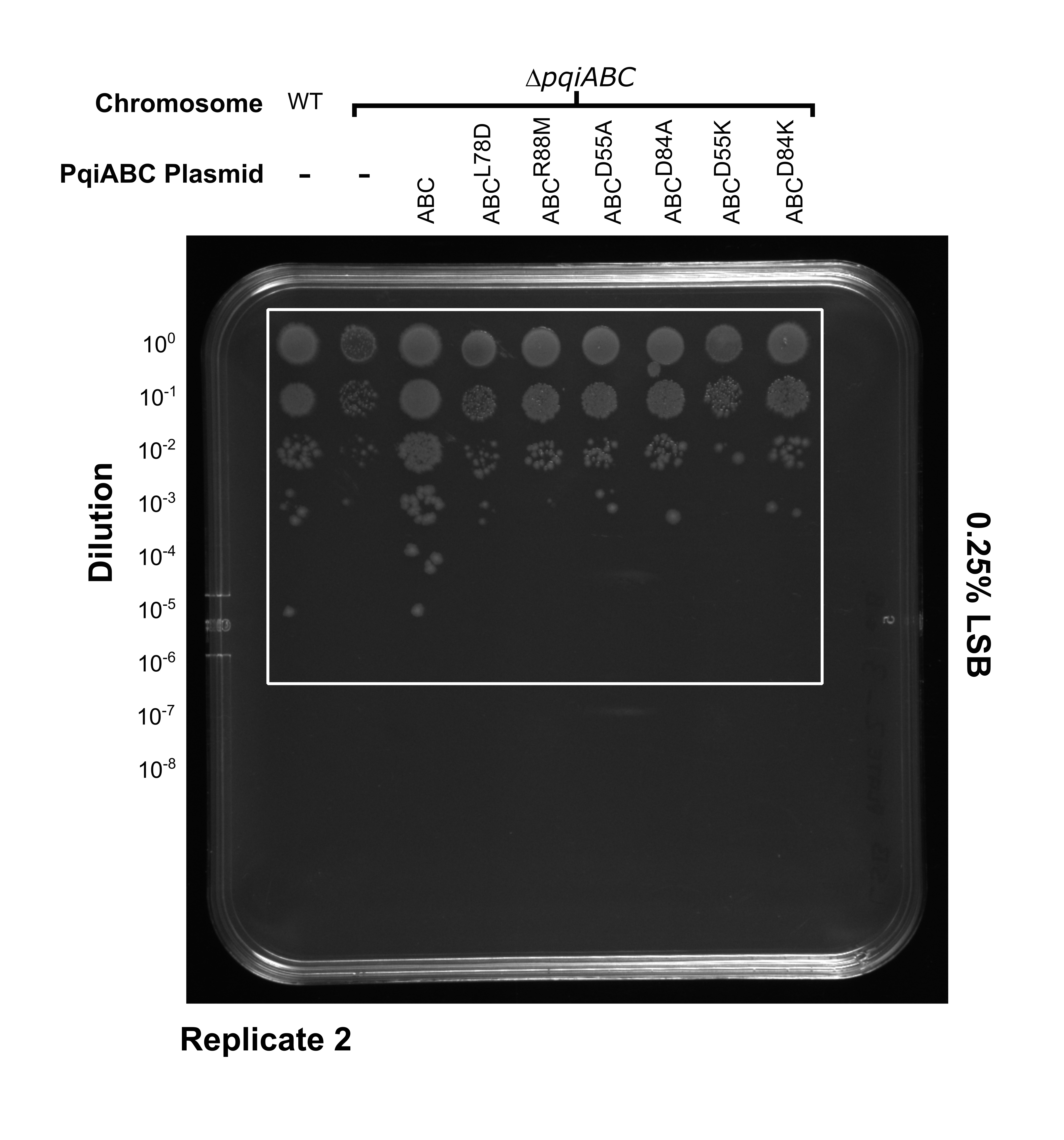

Supplement: Supplementary file 3 — Source Data Fig. 5 [file 44319_2023_14_MOESM3_ESM.zip › Source Data/Figure 5/5C/Figure 5C - 0.25% LSB replicates/Figure 5C - 0.25% LSB - Replicate 2.png]

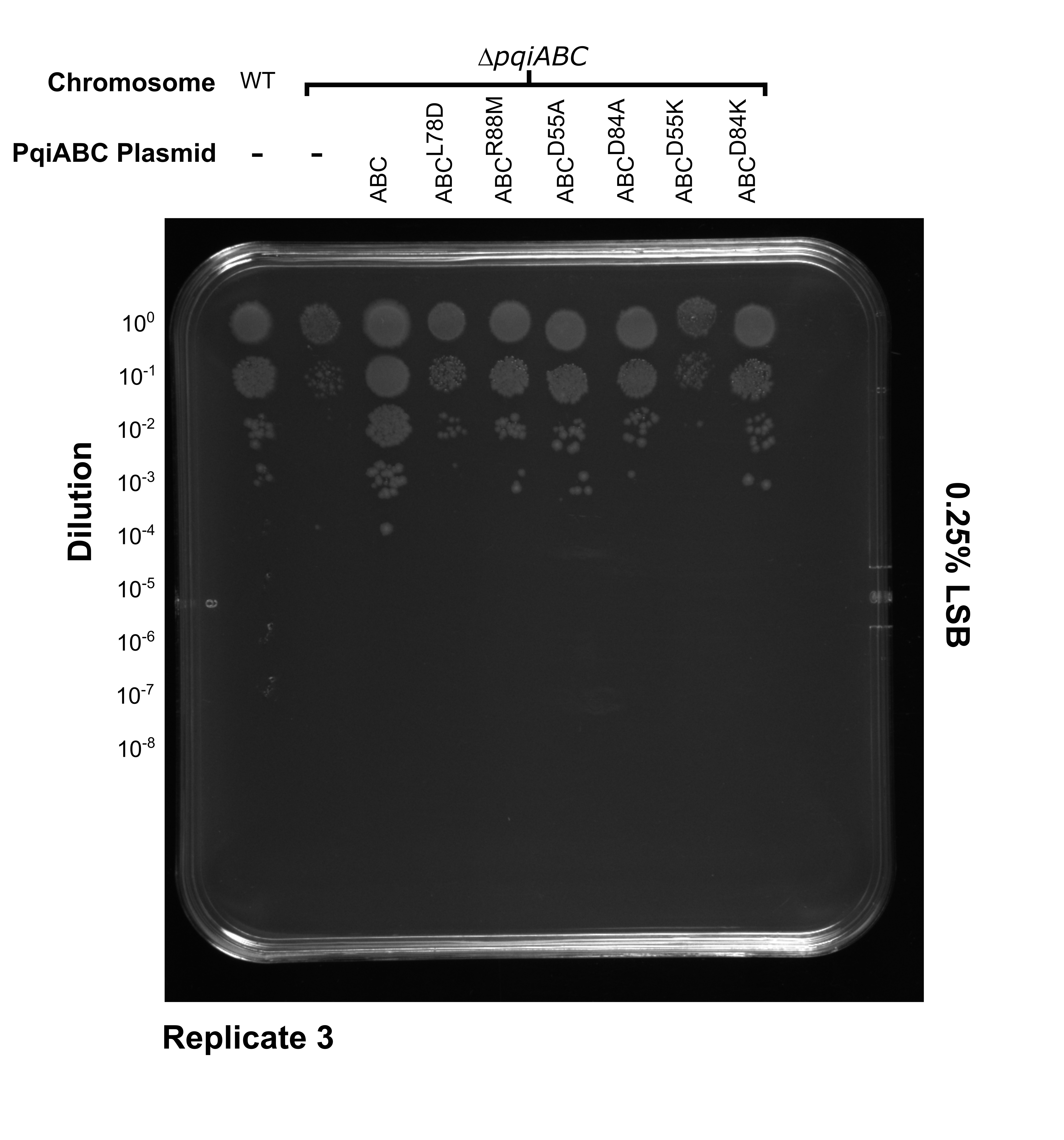

Supplement: Supplementary file 3 — Source Data Fig. 5 [file 44319_2023_14_MOESM3_ESM.zip › Source Data/Figure 5/5C/Figure 5C - 0.25% LSB replicates/Figure 5C - 0.25% LSB - Replicate 3.png]

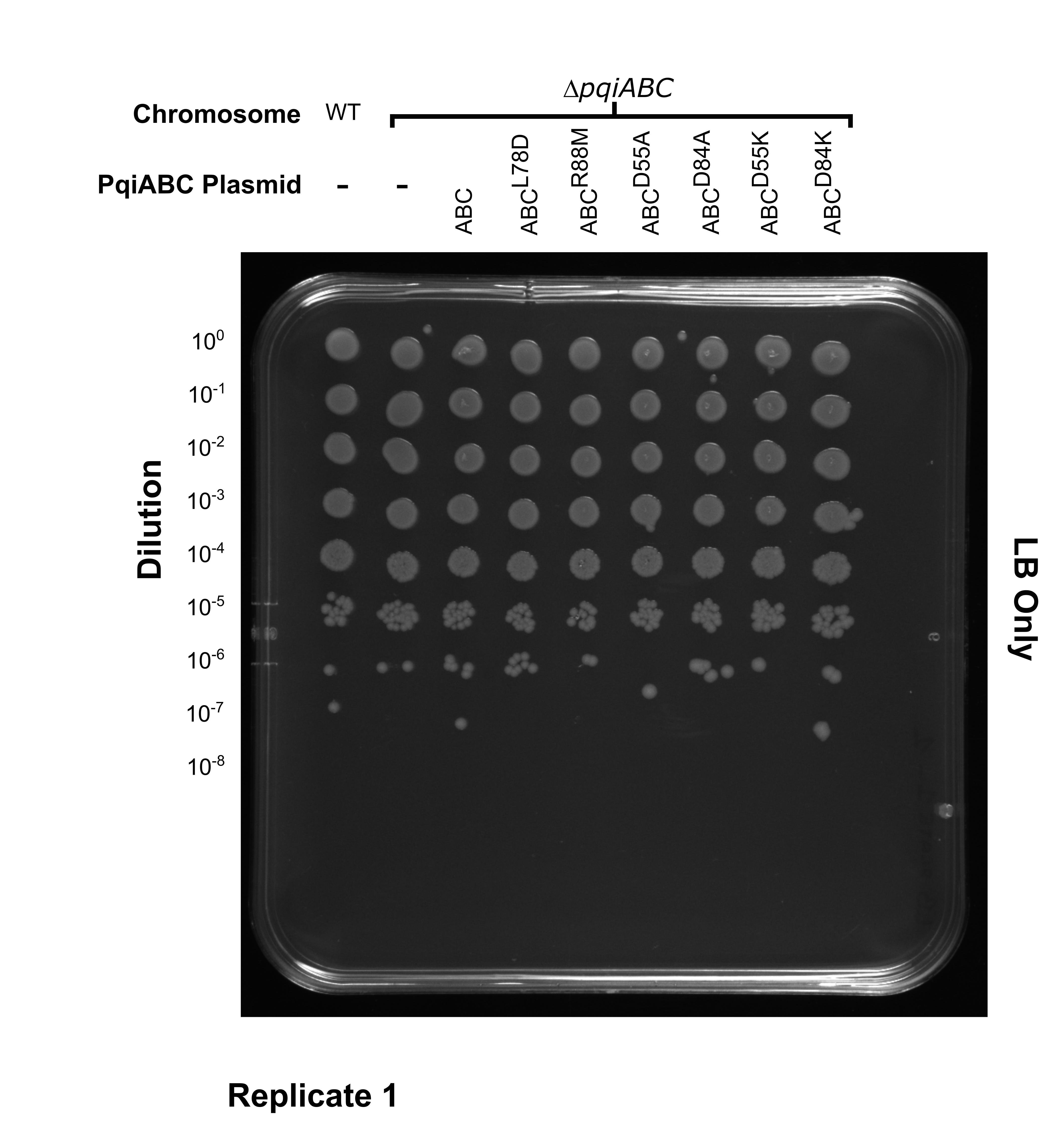

Supplement: Supplementary file 3 — Source Data Fig. 5 [file 44319_2023_14_MOESM3_ESM.zip › Source Data/Figure 5/5C/Figure 5C - LB replicates/Figure 5C - LB replicate 1.png]

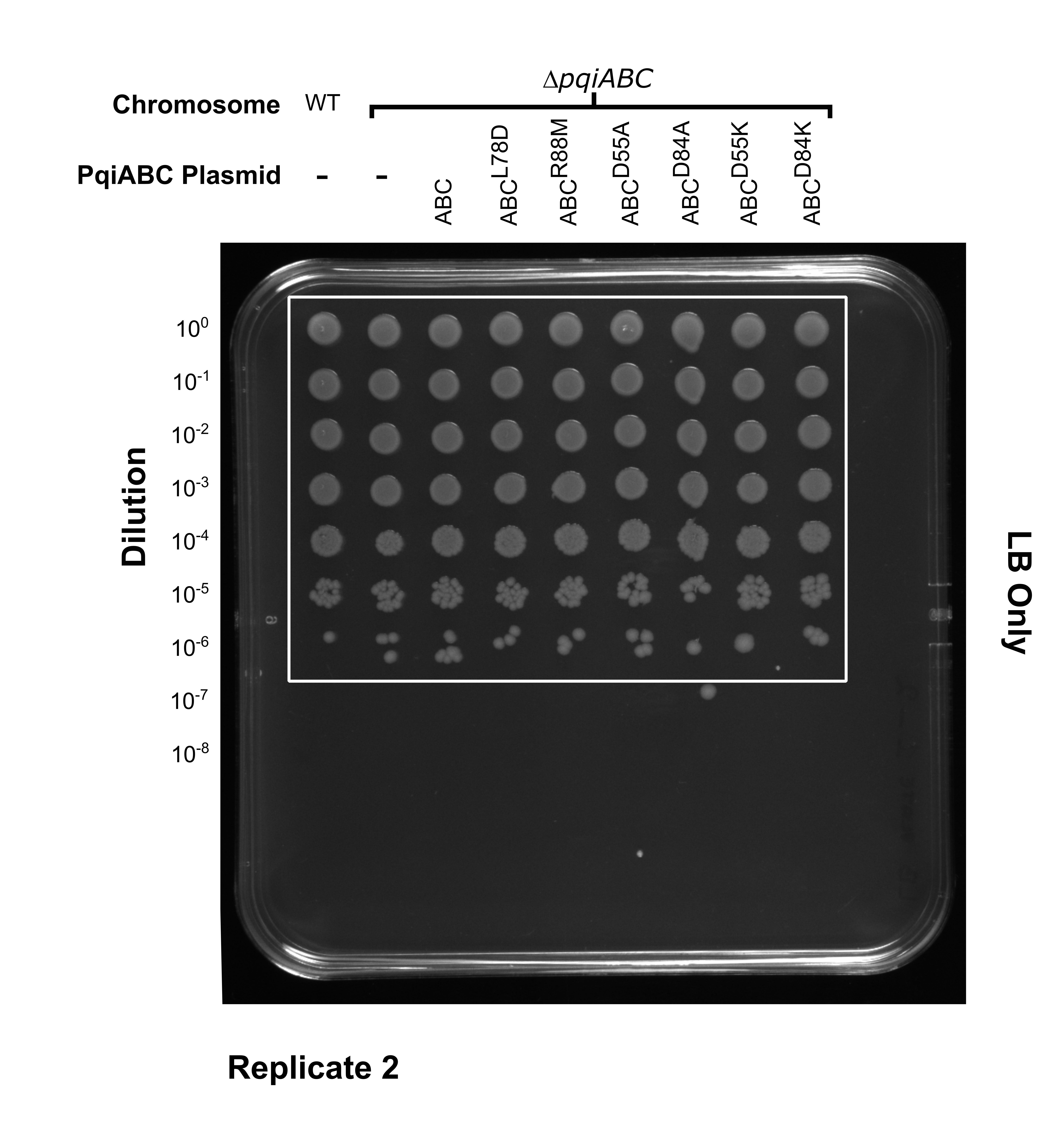

Supplement: Supplementary file 3 — Source Data Fig. 5 [file 44319_2023_14_MOESM3_ESM.zip › Source Data/Figure 5/5C/Figure 5C - LB replicates/Figure 5C - LB replicate 2.png]

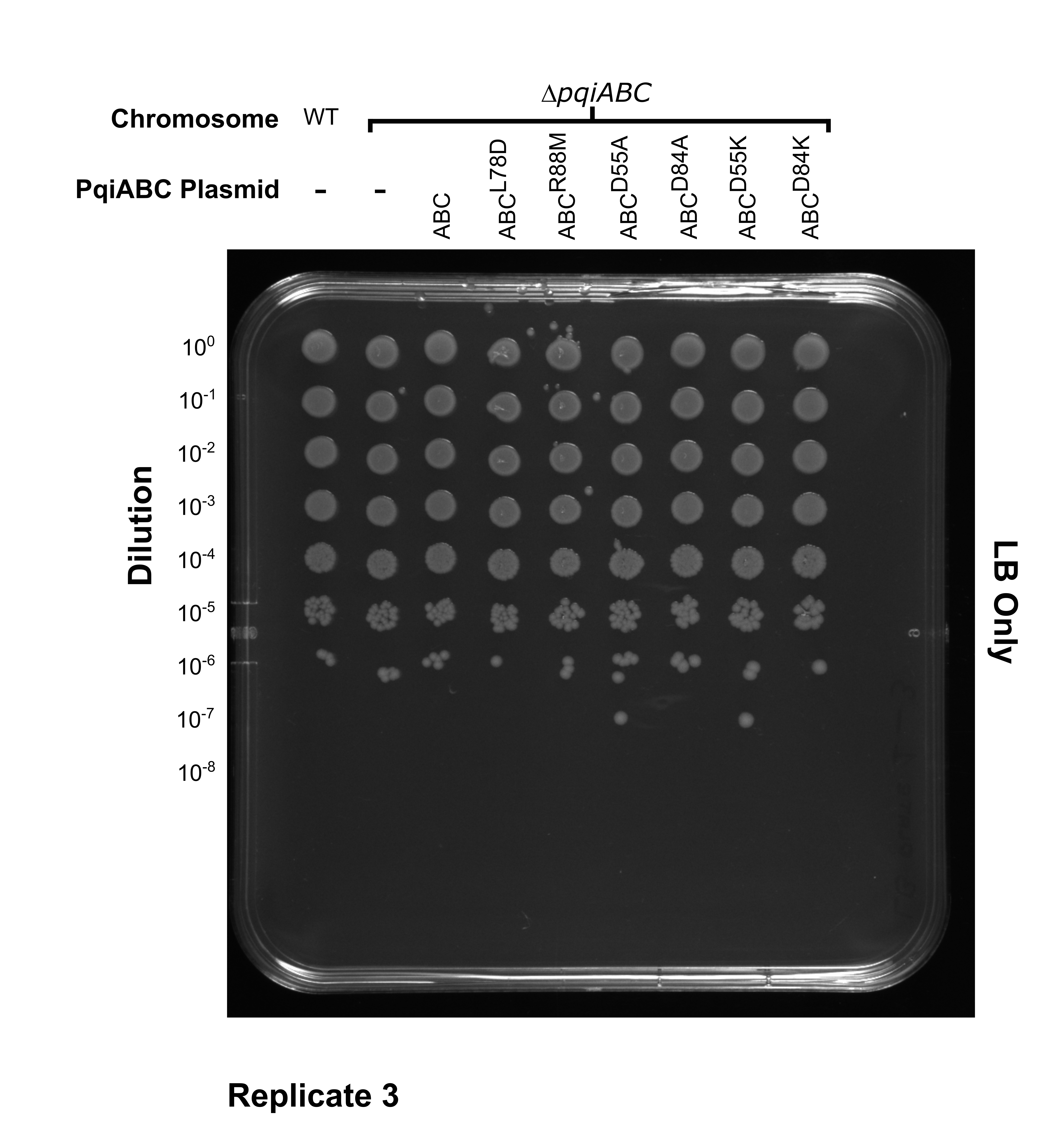

Supplement: Supplementary file 3 — Source Data Fig. 5 [file 44319_2023_14_MOESM3_ESM.zip › Source Data/Figure 5/5C/Figure 5C - LB replicates/Figure 5C - LB replicate 3.png]

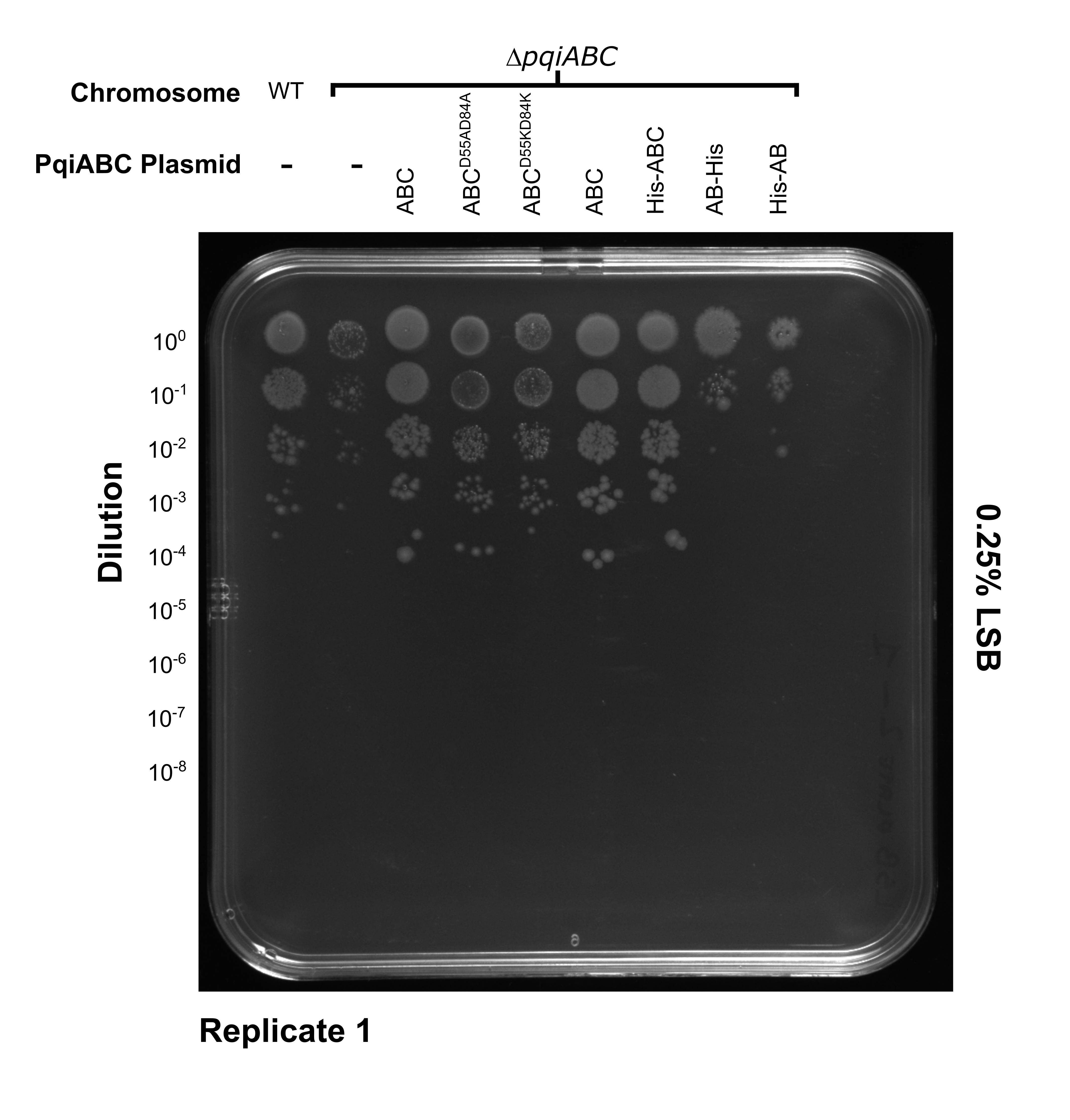

Supplement: Supplementary file 3 — Source Data Fig. 5 [file 44319_2023_14_MOESM3_ESM.zip › Source Data/Figure 5/5D/Figure 5D - 0.25% LSB replicates/Figure 5D - 0.25% LSB replicate 1.png]

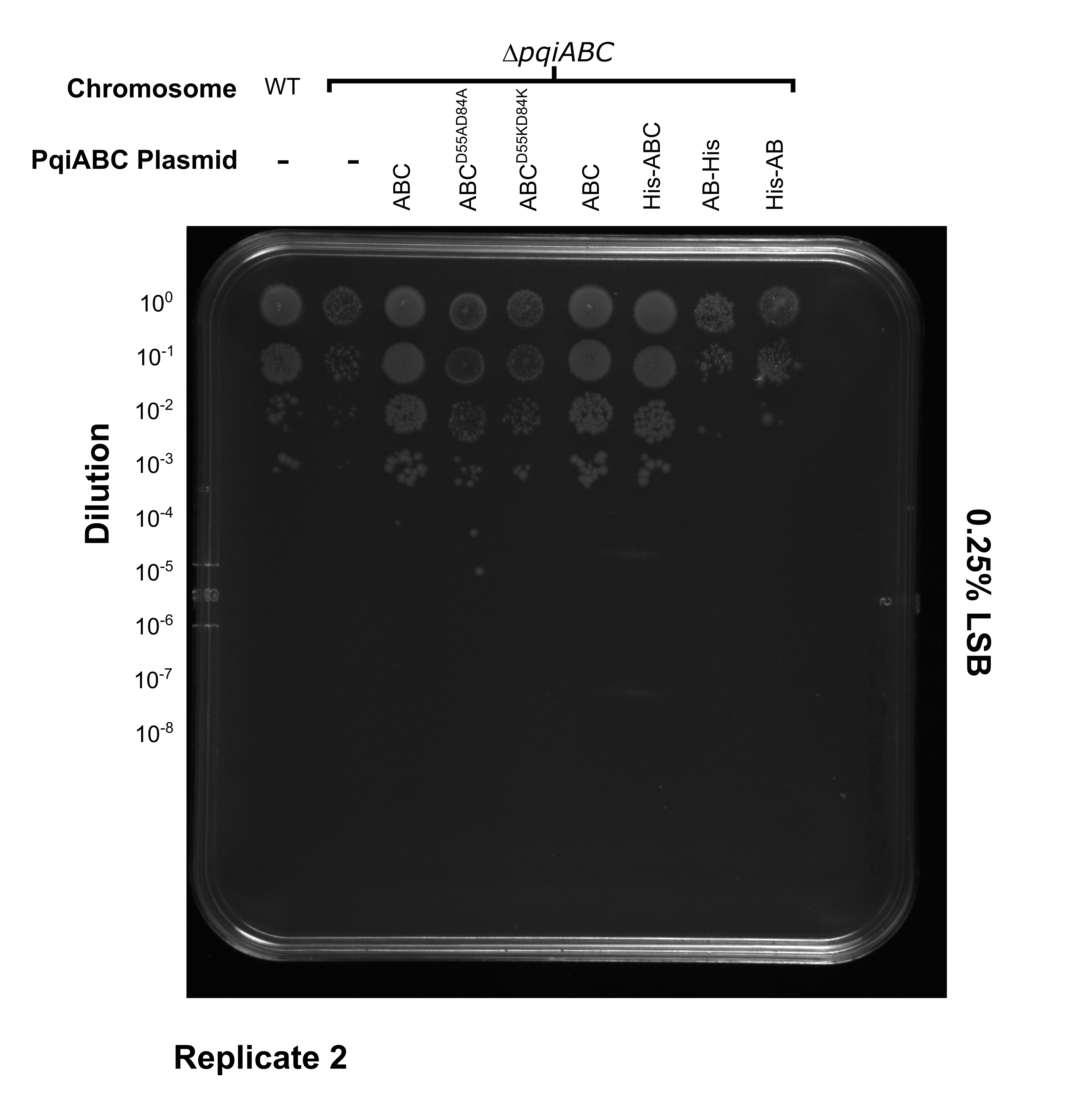

Supplement: Supplementary file 3 — Source Data Fig. 5 [file 44319_2023_14_MOESM3_ESM.zip › Source Data/Figure 5/5D/Figure 5D - 0.25% LSB replicates/Figure 5D - 0.25% LSB replicate 2.png]

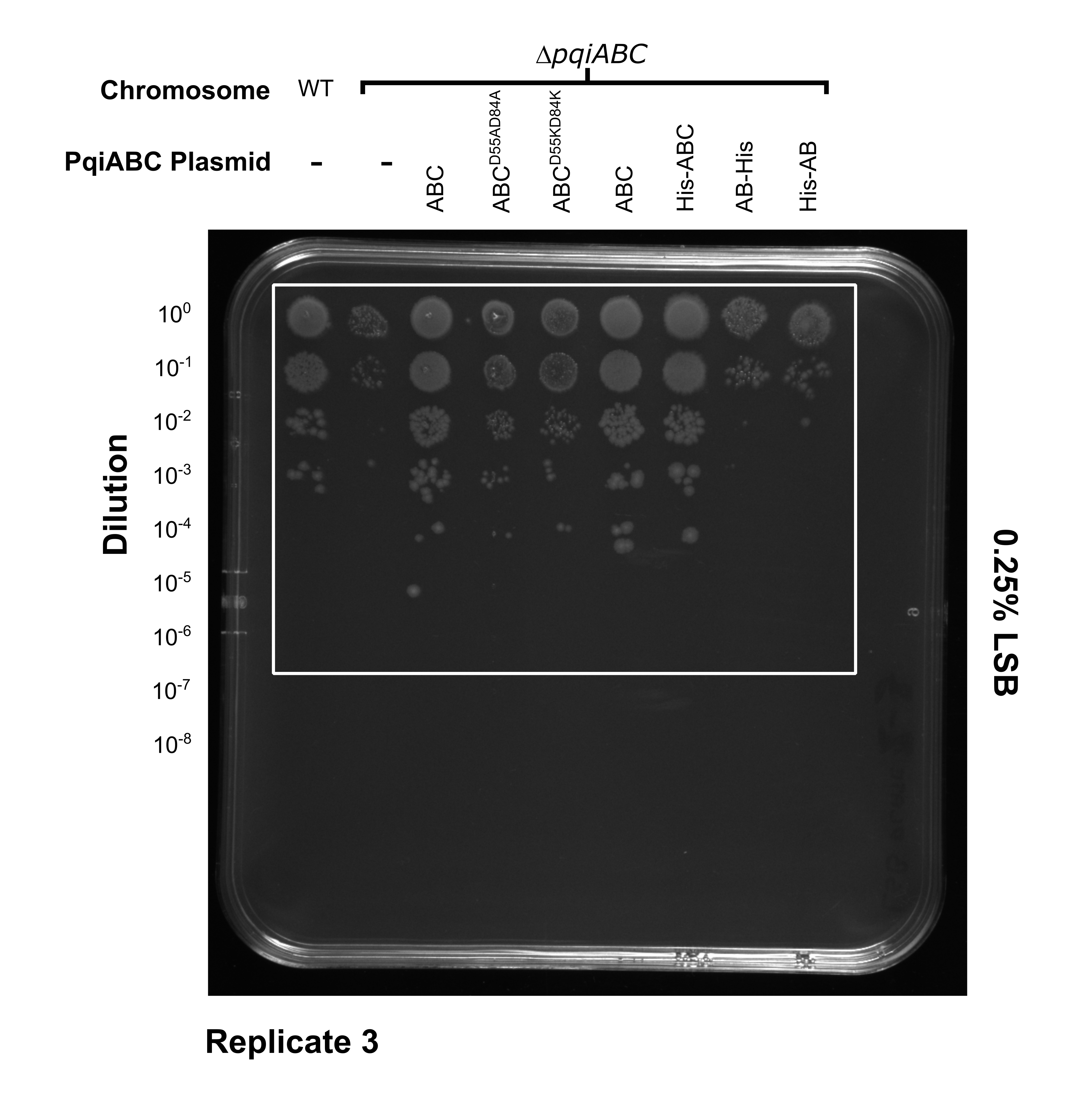

Supplement: Supplementary file 3 — Source Data Fig. 5 [file 44319_2023_14_MOESM3_ESM.zip › Source Data/Figure 5/5D/Figure 5D - 0.25% LSB replicates/Figure 5D - 0.25% LSB replicate 3.png]

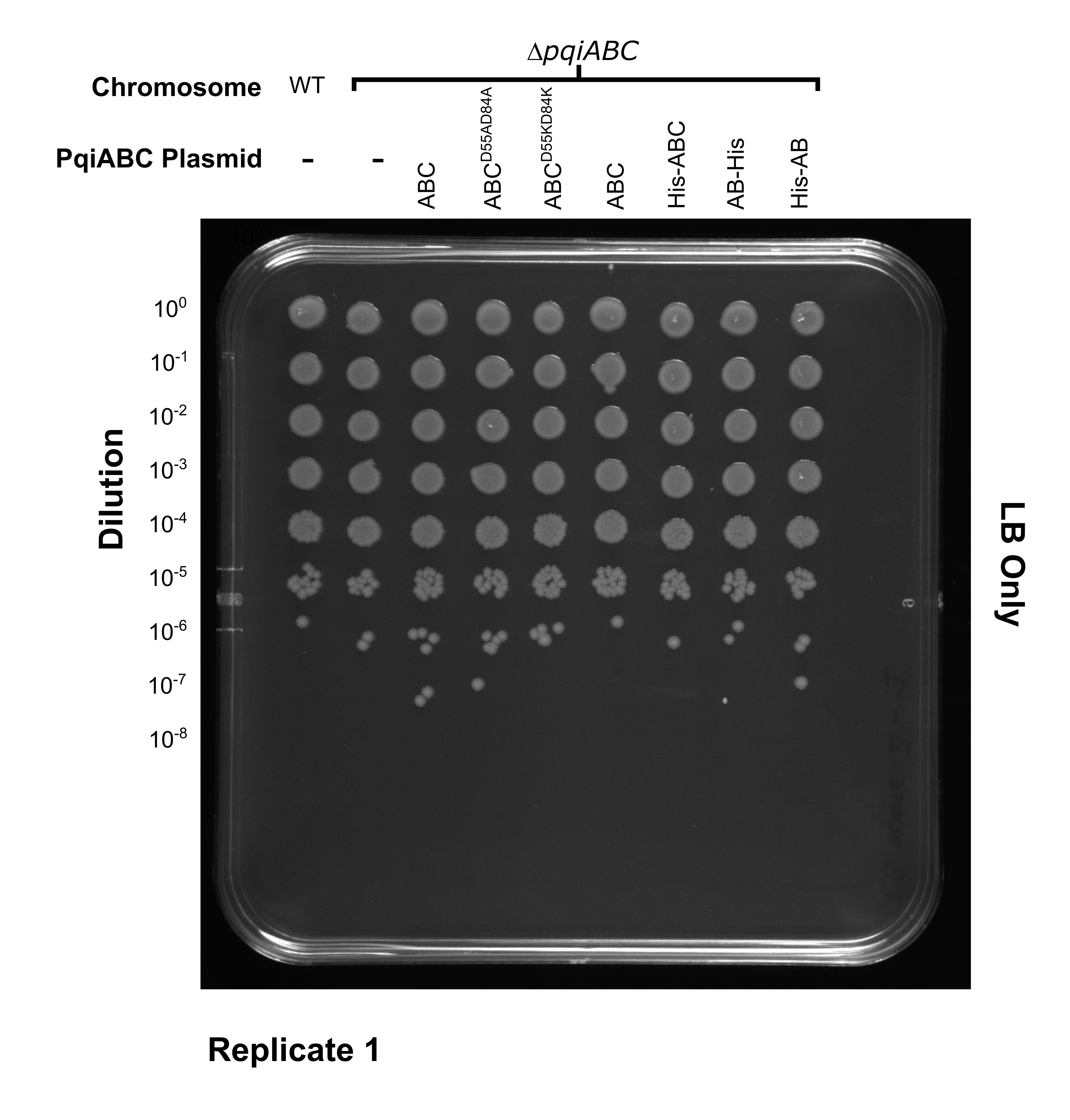

Supplement: Supplementary file 3 — Source Data Fig. 5 [file 44319_2023_14_MOESM3_ESM.zip › Source Data/Figure 5/5D/Figure 5D - LB replicates/Figure 5D - LB replicate 1.png]

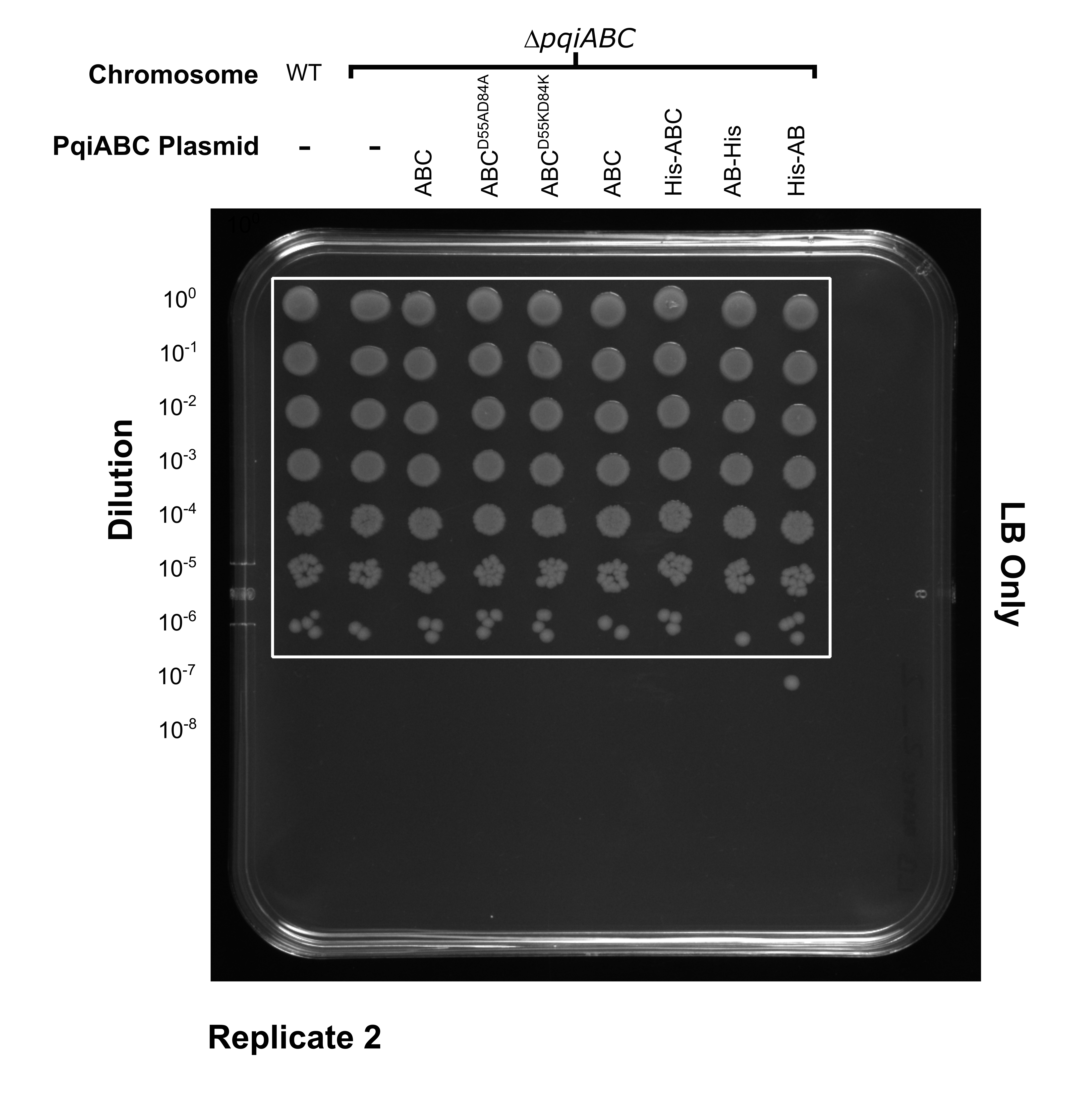

Supplement: Supplementary file 3 — Source Data Fig. 5 [file 44319_2023_14_MOESM3_ESM.zip › Source Data/Figure 5/5D/Figure 5D - LB replicates/Figure 5D - LB replicate 2.png]

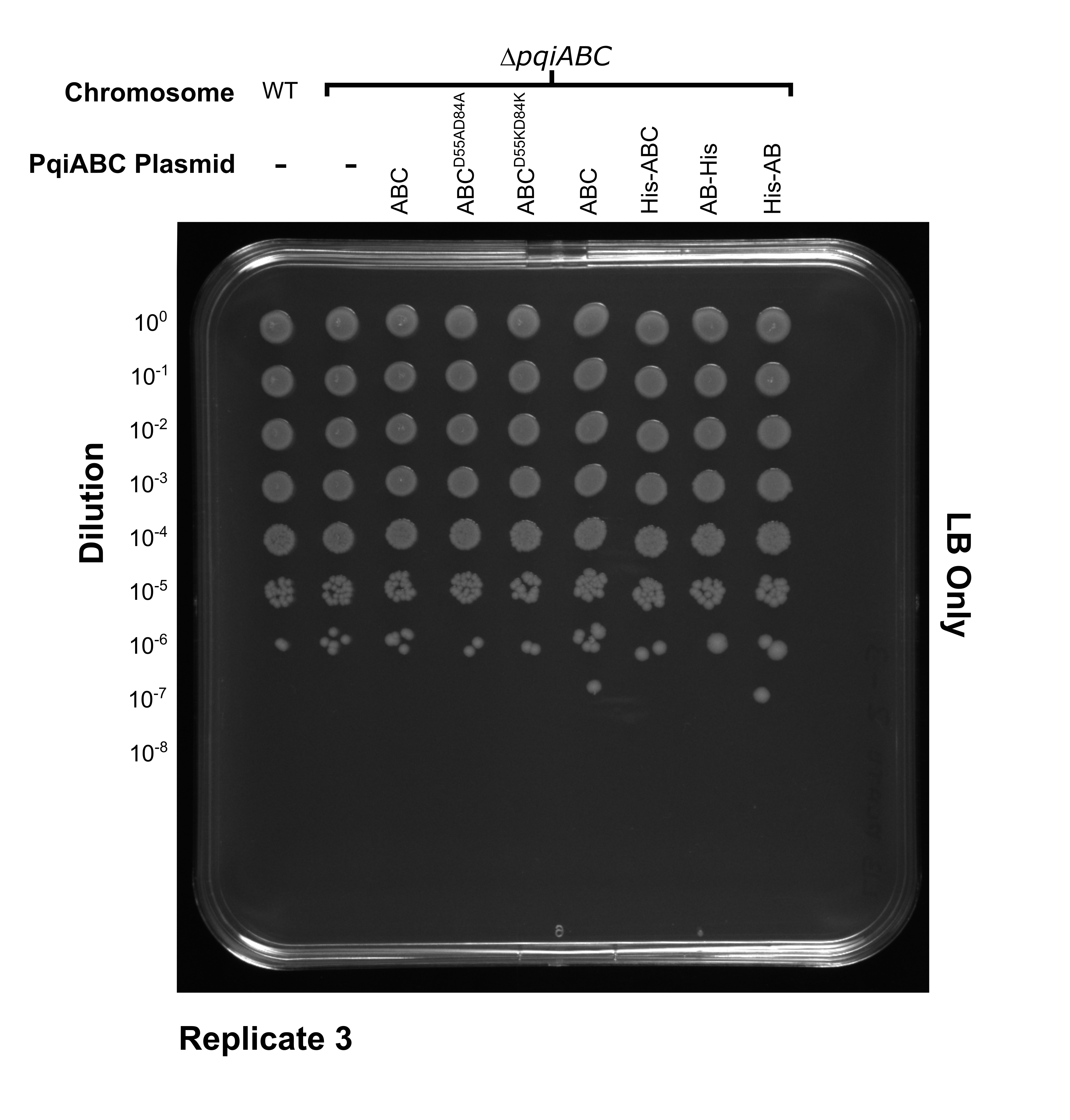

Supplement: Supplementary file 3 — Source Data Fig. 5 [file 44319_2023_14_MOESM3_ESM.zip › Source Data/Figure 5/5D/Figure 5D - LB replicates/Figure 5D - LB replicate 3.png]
